# Sprayable NIR/pH-responsive NSC/PF127 hydrogel loaded with MoO₂, ciprofloxacin, and FGF-2 for enhanced antibacterial and pro-regenerative infected wound healing

**DOI:** 10.1080/10717544.2026.2719063

**Published:** 2026-09-03

**Authors:** Zhirui Li, Mingxing Lei, Chen Chen, Shuaishuai Li, Guoqi Wang

**Affiliations:** a Department of Orthopaedics, Hainan Hospital of Chinese PLA General Hospital, Sanya, China; b National Clinical Research Center for Orthopedics, Sports Medicine & Rehabilitation, Beijing, China; c Department of Nursing, The First Medical Center of Chinese People's Liberation Army General Hospital, Beijing, China; d Senior Department of Pediatrics, Chinese People's Liberation Army (PLA) Hospital, Beijing, China

**Keywords:** Biofilm disruption, infected wound healing, MoO₂ nanoparticles, photothermal therapy, stimuli-responsive hydrogel, sustained growth factor delivery

## Abstract

Infected wounds represent a significant clinical burden owing to persistent infection, excessive inflammation, and impaired tissue regeneration. To overcome these limitations, we developed a sprayable *N*-succinyl chitosan/Pluronic F127 (NSC/PF127) hydrogel incorporating MoO₂ nanoparticles, ciprofloxacin, and fibroblast growth factor-2 (FGF-2). At body temperature, the hydrogel rapidly transitions from a solution to a gel and responds to both pH changes and near-infrared (NIR) irradiation. MoO₂ exhibits NIR-mediated photothermal activity, while CIP and FGF-2 are released in a sustained and pH-dependent manner, thereby maintaining antibacterial efficacy and growth factor bioactivity. *In vitro* studies demonstrated excellent cytocompatibility and significantly enhanced fibroblast and keratinocyte migration. The combined photothermal effect and antibiotic therapy effectively suppressed bacterial growth and disrupted established biofilms. In a rat model of *Staphylococcus aureus*-infected full-thickness wounds, the hydrogel composite significantly accelerated healing, reduced inflammation, promoted collagen deposition and angiogenesis, and enhanced tissue regeneration. Biochemical and gene expression analyses further indicated reduced oxidative stress and improved tissue regeneration. This multifunctional, sprayable hydrogel combines targeted antibacterial action with sustained pro-regenerative signaling, offering a promising strategy for further preclinical investigations of infected wound management.

## Graphical abstract

**Figure:**
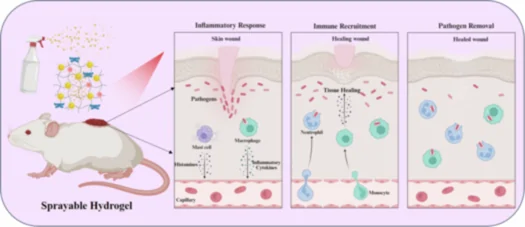

## 1. Introduction

Chronic wounds are a major healthcare challenge worldwide, particularly in the aging population and patients with diabetes or obesity, where impaired healing is often accompanied by persistent inflammation, recurrent infection, and delayed tissue regeneration (Falanga et al. 2022; Ghosh et al. 2022; Nazli et al. 2022). Biofilm formation, which hampers antibiotic penetration, shields bacteria from host immune responses and sustains the inflammatory microenvironment, worsens wound healing in the presence of persistent bacterial infection, especially methicillin-resistant Staphylococcus aureus (MRSA) (Xiao et al. 2021; Li et al. 2023). Common therapeutic strategies such as systemic antibiotics, surgical debridement, and topical growth factor delivery generally present limited clinical outcomes because of low drug retention, rapid degradation of bioactive molecules, drug resistance, and inability to provide a long-term regenerative microenvironment (Ghosh et al. 2022; Nazli et al. 2022; Xiao et al. 2021; Li et al. 2023).

Hydrogel-based wound dressings have become promising biomaterials, as they provide a moist three-dimensional matrix supporting cell migration while allowing local and sustained delivery of therapeutic agents (Xu and Pu 2021; Zhang et al. 2021). Among these systems, stimuli-responsive hydrogels, especially those responsive to NIR light, have received significant attention because of their ability to enable external therapeutic activation and on-demand drug release. The incorporation of photothermal nanomaterials into hydrogel matrices has been demonstrated to improve antibacterial efficacy, disrupt bacterial biofilms, and modulate the inflammatory microenvironment for wound healing (Pitarresi et al. 2023; Hlushko et al. 2024; Di Domenico et al. 2022; Wu et al. 2021). Fibroblast growth factors such as FGF-2 are also critical for fibroblast proliferation, angiogenesis, and extracellular matrix remodeling. However, their therapeutic application is limited by rapid degradation and poor retention at the wound site.

Although sprayable hydrogels, photothermal nanomaterials, localized antibiotic delivery systems, and FGF-2-loaded wound dressings have been reported separately in previous studies, relatively few investigations have systematically integrated these therapeutic functions into a single platform and comprehensively evaluated their combined performance against the multiple pathological events involved in infected wound healing. Current wound dressings are still facing challenges in controlling bacterial infection, biofilm formation, oxidative stress, inflammation, and impaired tissue regeneration simultaneously (Chen et al. 2022; Li et al. 2021; Park et al. 2020; Liu et al. 2024; Yao et al. 2025; Fan et al. 2025).

Therefore, in the present study, we designed an N-succinyl chitosan/Pluronic F127-based sprayable NIR/pH-responsive hydrogel containing MoO_2_ nanoparticles, ciprofloxacin, and FGF-2 as adjunct therapeutic components. The present work aims to rationally integrate well-established therapeutic strategies into a multifunctional platform capable of combining localized antibacterial therapy, NIR-responsive photothermal treatment, sustained growth factor delivery, and tissue regenerative support rather than developing new constituent materials. The developed hydrogel was systematically investigated *in vitro* and *in vivo* for its physicochemical properties, antibacterial activity, antioxidant capacity, cytocompatibility, regenerative potential, and wound healing efficacy for its potential as a multifunctional dressing for infected wound healing.

## 2. Materials and methods

### 2.1. Materials used

Pluronic F127 (PEO_100_–PPO_65_–PEO_100_, average M_n_ ≈ 12,600 Da), chitosan (CS) (degree of deacetylation 75 to 85%), succinic anhydride (SA), molybdenum(IV) oxide (MoO_2_; assay ≥ 99.5%), ciprofloxacin hydrochloride (CIP), FGF-2, (recombinant human, ≥ 98% purity) 2,2-diphenyl-1-picrylhydrazyl (DPPH), phosphate-buffered saline tablets, dimethyl sulfoxide (DMSO), and sodium hydroxide were procured from Sigma-Aldrich (USA). Fetal bovine serum (FBS), Dulbecco's Modified Eagle Medium (DMEM), penicillin–streptomycin (1%), and trypsin-EDTA were obtained from Shanghai BasalMedia Technologies Co., Ltd. (Shanghai, China). The murine fibroblast cell line (L929), human keratinocyte cell line (HaCaT), and mouse embryonic fibroblast cell line (NIH 3T3) were purchased from Ubigene (China). All reagents were of analytical grade and were used without further purification. Ultrapure de-ionized (DI) water was used throughout the experiments.

### 2.2. Synthesis of N-succinyl chitosan (NSC)

NSC-1 was synthesized according to the method reported elsewhere with slight modifications (Wei et al. 2025). In brief, 0.5 g of CS was dissolved in 50 mL of acetic acid (2% v/v) solution and stirred for an hour at 30 °C to ensure complete dissolution. Separately, SA was dissolved in 25 mL of methanol at 50 °C. The SA solution corresponding to a 1:1 molar ratio with the amino group of CS was slowly added to the CS solution under constant stirring. After complete addition, the reaction mixture was maintained at 30 °C with constant stirring for 24 h. After completion of the reaction, the mixture was neutralized to pH 7 with 1 M NaOH and stirred for 2 h at room temperature. The neutralized product was precipitated using cold ethanol and collected via centrifugation. The resulting precipitate was redissolved in DI water and stirred for 18 h to remove the unreacted reactants. The pH of the mixture was adjusted to 12 by adding 1 M NaOH, and the mixture was left undisturbed for 24 h. The final product was precipitated with cold ethanol, centrifuged, and washed several times with ethanol and acetone. The purified NSC-1 was finally dried in an air oven at 50 °C for 5 h.

### 2.3. Preparation of NSC/PF127 sprayable hydrogel

The NSC/PF127 Sprayable Hydrogel formulation was prepared using a cold-mixing method to avert premature gelation and ensure uniform blending. The pre-prepared NSC solution (0.25 wt %) was cooled to 4 °C before blending. Meanwhile, the PF127 solution (20 wt%) was also prepared and kept at the same temperature. The two chilled solutions were mixed by stirring at 4 °C. Stirring was continued until the mixture appeared clear and homogeneous. The resulting mixture was kept at 4 °C overnight to allow complete dissolution and hydration of PF127. The final product was a uniform, sprayable hydrogel solution that underwent gelation at body temperature.

### 2.4. Incorporation of MoO_2_, FGF-2 and CIP on hydrogel

To incorporate the nanoparticles, 50 mg of MoO_2_ was dispersed in 10 mL of NSC/PF127 solution and sonicated for 5 min to achieve a uniform distribution. CIP (2 mg/mL) was added to the above dispersion and allowed to equilibrate to promote interactions with the hydrogel matrix for 2 h. Finally, FGF-2 (100 ng/mL) was gently added and mixed at 4 °C for 30 min to obtain the final precursor solution.

### 2.5. Characterization of hydrogel

#### 2.5.1. Physicochemical characterisations

FT-IR spectra (4000–400 cm^−1^, Bruker Alpha II) of dried KBr pellet samples with CS, NSC, PF127, NSC/PF127, MoO_2,_ CIP, FGF-2 and the composite hydrogel were analyzed to identify the characteristic bands for amide, ether, and Mo‒O bonds, thereby confirming the successful incorporation of MoO_2_, FGF-2, and CIP into the hydrogel matrix. ^1^H NMR spectra were obtained using a Bruker Avance 400 MHz spectrometer in D_2_O at 25 °C. The characteristic –CH₂–CH₂–COOH proton signals were used to confirm the successful N-succinylation of CS, and the peak intensities were used to determine the degree of succinyl substitution.

#### 2.5.2. Rheological measurements and sol–gel transition

The viscoelastic properties and thermosensitive behavior of the hydrogel were evaluated using a rotational rheometer (Anton Paar MCR 302, Austria) equipped with a 25 mm parallel-plate geometry. The hydrogel samples (1 mL) were placed between the two plates, and oscillatory measurements were carried out at a frequency of 1 Hz and a strain of 1% while the temperature was steadily increased from 10 °C to 50 °C at a rate of 2 °C min^−1^. The sol‒gel transition temperature (*T*_gel_) was determined at the point where the storage modulus (*G*ʹ) exceeded the loss modulus (*G*").

#### 2.5.3. Swelling ratio measurement

Hydrogel discs (diameter 10 mm, thickness 2 mm) were initially dried and accurately weighed (Wd) to determine their dry weight. The samples were then immersed in 10 mL of PBS buffer (pH 7.4) and incubated at 37 °C. At the specified time intervals (1, 3, 5, 7, and 14 days), the hydrogels were removed and gently wiped with tissue paper to remove surface moisture, and the measured weight was recorded as the swollen weight (*W*_s_). The swelling ratio was calculated as the ratio of swollen weight to dry weight (*W*_s_/W_d_). Each measurement was performed in triplicate for accuracy and reproducibility.

$$\mathrm{Swelling}\;\mathrm{ratio}\;\left(\%\right)=\frac{W_{s}}{W_{d}}\times 100.$$

#### 2.5.4. Water retention studies

Hydrogel samples were immersed in PBS buffer (pH 7.4) and incubated at 37 °C for 24 h to reach equilibrium. After swelling, the hydrogel samples were removed from the buffer, gently wiped with tissue paper to remove surface water, and weighed to obtain the initial weight (*W*_i_). Then the samples were exposed to normal laboratory conditions and weighed at regular intervals (1, 3, 5, 7, and 14 h) to measure weight loss. The water retained in the sample at each time point was measured by comparing the weight at that time (W_t_) with the initial weight (*W*_i_). Measurements were performed in triplicate to ensure reliability.

$$\mathrm{Water}\;\mathrm{retention}\;(\%)\;=\frac{W_{t}}{W_{i}}\times 100.$$

#### 2.5.5. Degradation measurements

Hydrogel discs (diameter 10 mm, thickness 2 mm) were accurately weighed (W_0_), immersed in 10 mL of PBS (pH 7.4 and pH 5.5), and incubated at 37 °C. At predetermined time points (1, 3, 5, 7, and 14 days), samples were withdrawn, gently washed, lyophilized and weighed (*W*_t_). The percentage of degradation was measured using the following equation:

$$\text{Degradation rate}\;(\%)=\frac{W_{0}-W_{t}}{W_{0}}\times 100.$$

All measurements were performed in triplicate.

#### 2.5.6. *In vitro* drug release studies

CIP and FGF-2 release from the hydrogel composite were evaluated in PBS buffer at pH 5.5 and 7.4 to mimic normal and infected wound environments. Hydrogel samples containing the equivalent of 2 mg of CIP and the corresponding amount of FGF-2 were placed into a dialysis bag (MWCO 12–14 kDa) and immersed in 30 mL of buffer at 37 °C with gentle shaking (100 rpm). At the specific time point, 2 mL of the release medium was collected and replaced with fresh PBS. The concentration of the drug was measured using the UV–Vis spectrophotometer (*λ* = 277 nm). The release of FGF-2 was evaluated using an ELISA following the manufacturer's protocol to ensure sensitivity and preservation of protein integrity.

To investigate the drug release mechanism, the cumulative release data of CIP and FGF-2 were analyzed using five commonly employed kinetic models, namely, the zero-order, first-order, Higuchi, Korsmeyer–Peppas, and Hixson–Crowell models. Linear regression analysis was performed using the corresponding linearized equations for each kinetic model. The goodness of fit was evaluated by comparing the correlation coefficient (*R* ^2^), adjusted *R* ^2^, root mean square error (RMSE), and corresponding release rate constant (k). For the Korsmeyer–Peppas model, the release exponent (*n*) was additionally determined to characterize the drug release mechanism. Because the Korsmeyer–Peppas model applies only to the initial stage of drug release, an additional early-time release study with closely spaced sampling intervals during the initial burst-release phase was performed to obtain sufficient data points for reliable kinetic modeling.

#### 2.5.7. Photothermal performance

The photothermal behavior of MoO₂-loaded hydrogels was assessed under 808-nm NIR irradiation (0.75 W cm^−2^ for 7 min). The temperature elevation was monitored using an infrared thermal camera at 10 s intervals. Photothermal stability was evaluated based on maximum temperature elevation, reproducibility across cycles, and absence of signal decay.

### 2.6. *In vitro* antibacterial activity evaluation

#### 2.6.1. Minimum inhibitory concentration (MIC) assay

MICs were determined via broth microdilution *against E. coli, P. aeruginosa,* and *MRSA*. Bacteria were grown to the logarithmic phase and diluted in LB broth to approximately 1 × 10^6^ CFU mL^−1^. Serial two-fold dilutions of CIP, Gel, Gel + CIP, Gel@MoO₂, and Gel@MoO₂ + CIP + FGF-2 were prepared in 96-well plates, followed by the addition of a bacterial suspension. For MoO₂-containing groups, selected wells were exposed to NIR (808 nm, 0.75 W cm^−2^) for 5 min, while parallel samples were maintained under identical conditions without irradiation. The plates were incubated at 37 °C for 24 h, and bacterial growth was assessed by visual inspection and by measuring the optical density at 570 nm. The MIC was defined as the lowest concentration at which no visible growth was observed. All the experiments were carried out in triplicate.

#### 2.6.2. *In vitro* CFU assay

The *in vitro* antibacterial activity of the hydrogel formulations was assessed using a colony-forming unit (CFU) assay against *E. coli, P. aeruginosa,* and *MRSA*. The bacterial suspensions (1 × 10^6^ CFU mL^−1^) were then treated with PBS (control), CIP, Gel, Gel + CIP, Gel@MoO₂, and Gel@MoO₂ + CIP + FGF-2 at various concentrations. The samples containing MoO₂ were either exposed to 808 nm NIR laser irradiation (0.75 W cm^−2^, 5 min) or kept under identical non-irradiated conditions. Following incubation at 37 °C, treated bacterial suspensions were serially diluted with PBS, and 100 μL aliquots were spread onto LB agar plates. The plates were incubated at 37 °C for 24 h, after which the bacterial colonies were photographed and quantified using ImageJ software. All experiments were performed in triplicate, and antibacterial activity was determined by comparing viable CFU counts with those of the PBS-treated control group.

#### 2.6.3. *In vitro* antibiofilm assay

The antibiofilm activity of the hydrogel formulations against *E. coli, P. aeruginosa*, and *MRSA* was evaluated using a crystal violet staining assay. Bacterial suspensions (1 × 10^7^ CFU mL^−1^) prepared in tryptic soy broth were added to 96-well plates and incubated at 37 °C for 48 h to allow biofilm formation. After removal of non-adherent cells and washing with PBS, the established biofilms were treated with PBS (control), CIP, Gel, Gel + CIP, Gel@MoO₂, and Gel@MoO₂ + CIP + FGF-2 at predetermined concentrations. For groups containing MoO₂, NIR irradiation (808 nm, 0.75 W cm^−2^, 5 min) was applied. After an additional 24 h of treatment, the biofilms were fixed, stained with 0.1% crystal violet, and the bound dye was solubilized with ethanol. The absorbance was measured at 570 nm to quantify the biofilm biomass. All the assays were conducted in triplicate, and biofilm inhibition was calculated by comparing the treated group with the untreated control group.

#### 2.6.4. Zone of inhibition assay

The antibacterial activity of the hydrogel formulations was evaluated via an agar diffusion assay against *E. coli, P. aeruginosa,* and *MRSA*. The bacterial cultures were grown to the exponential phase, adjusted to ~1 × 10^8^ CFU mL^−1^, and incorporated into molten nutrient agar (final concentration ~1 × 10^6^ CFU mL^−1^), which was poured onto agar plates. Sterile wells were formed after solidification and were filled with PBS (control), CIP, Gel, Gel + CIP, Gel@MoO₂, Gel@MoO₂ + CIP + FGF-2, and corresponding NIR-treated samples. NIR irradiation was applied at 808 nm (0.75 W cm^−2^, 5 min) where applicable. The plates were incubated at 37 °C for 24 h, after which the inhibition zone diameters were measured to assess antibacterial efficacy. All experiments were performed in triplicate.

#### 2.6.5. Confocal laser scanning microscopy (CLSM) analysis of bacterial viability

Bacterial viability within MRSA biofilms after different treatments was qualitatively evaluated using acridine orange/ethidium bromide (AO/EB) dual staining. MRSA biofilms were treated with PBS (control), Gel, CIP, Gel@MoO₂ + CIP + FGF-2 (NIR−), or Gel@MoO₂ + CIP + FGF-2 (NIR+). For the NIR group, biofilms were irradiated with an 808 nm laser (0.75 W cm^−2^, 5 min). Following treatment, the biofilms were gently washed with PBS and stained with an AO/EB solution for 10 min in the dark at room temperature. After removing excess stain by washing with PBS, the stained biofilms were immediately observed using a confocal laser scanning microscope (CLSM; Carl Zeiss LSM 710, Germany). Green fluorescence indicated viable bacteria, whereas red fluorescence represents membrane-compromised/non-viable bacteria. The AO/EB staining assay was performed solely to provide a qualitative visualization of bacterial viability and to corroborate the antibiofilm activity observed in the crystal violet assay; no quantitative analysis of live/dead bacterial populations was performed.

### 2.7. *In vitro* studies

#### 2.7.1. Cell viability

The cytocompatibility of the hydrogel samples was studied using L929, NIH 3T3, and HaCaT cells. In a 96-well plate, cells were seeded (1 × 10^4^ cells/well) and cultured in DMEM supplemented with 10% FBS and 1% penicillin–streptomycin at 37 °C under 5% CO₂. After 24 h, the medium was replaced with extracts of the hydrogel and drug samples. For NIR-responsive evaluation, Gel@MoO₂ + CIP + FGF-2 hydrogel extract was irradiated with an 808-nm laser (0.75 W cm^−2^, 5 min) before incubation. After 48 h of incubation, 10 μL of CCK-8 reagent was added to each well, and incubated for an additional 2 h. The absorbance was measured at 450 nm using a microplate reader. Cell viability was measured as a percentage of the control groups. A positive cytotoxic control (e.g. Triton X-100) was not included in this study, as the primary objective was to compare the relative cytocompatibility among formulations. This limitation has been acknowledged and will be addressed in future studies.

#### 2.7.2. Live and dead assay

To visualize cell viability, a live/dead assay was performed using AO/EB staining. The cells seeded on the coverslips were treated with the hydrogel and drug samples for 24 h. NIR stimulation (808 nm, 0.75 W cm^−2^, 5 min) was applied to the Gel@MoO₂ + CIP + FGF-2 group. The cover slips were then washed with PBS and stained with a 5 μg/mL AO/EB mixture for 5 min. The stained cells were observed under a fluorescence microscope (Leica DMi8, Germany). Live cells emit green fluorescence, and dead cells emit orange‒red fluorescence.

#### 2.7.3. Scratch wound migration assay

L929, NIH 3T3, and HaCaT cells were grown in 6-well plates until they reached near confluence. A sterile 200 μL pipette tip was used to make a uniform scratch on the plates, and the cells were treated with hydrogel samples in serum-free medium with NIR irradiation (0.75 W cm^−2^, 5 min) applied to the Gel@MoO₂ + CIP + FGF-2 hydrogel group at 0 h. The wound closure was photographed at 0 and 24 h using an inverted microscope. The percentage of wound closure was measured via ImageJ software using the following equation:

$$\text{Wound closure}\;(\%)=\frac{A_{0}-A_{t}}{A_{0}}\times 100,$$

where *A*_0_ and A_t_ are the wound areas at 0 h and 24 h, respectively.

#### 2.7.4. Intracellular ROS assay

The intracellular concentration of ROS was determined by means of DCFH-DA fluorescence analysis. The L929, NIH 3T3, and HaCaT cell lines were plated into a 24-well plate with 5 × 10^4^ cells in each well. The cells were maintained under conventional culture conditions (37 °C; 5% CO₂) for 24 h to allow cell attachment. Oxidative stress was induced in these cells by adding 200 µM hydrogen peroxide (H₂O₂) to the culture medium for 2 h. After that, the culture medium was aspirated, and the cells were washed with PBS. For the Gel@MoO₂ + CIP + FGF-2 group, NIR irradiation was applied at the beginning of the treatment period. The NIR irradiation parameters (0.75 W cm^−2^ for 5 min) were chosen according to preliminary optimization and previous reports for effective photothermal stimulation while maintaining thermal safety and minimizing damage to surrounding healthy tissues (Zhang et al. 2021; Yang et al. 2021; Yao et al. 2025). The cells were further incubated for 24 h. After treatment, the cells were washed with PBS and incubated with 10 μM DCFH-DA at 37 °C for 30 min in the dark. The excess dye was subsequently removed by washing with PBS. Fluorescence images, which represent the ROS level inside the cells, were acquired via fluorescence microscopy (Leica DMi8, Germany). The quantification of the fluorescence intensity was done using ImageJ software, and the ROS level inside the cells was represented as the normalized fluorescence intensity relative to that of the control group.

#### 2.7.5. Evaluation of the biological activity of released FGF-2

The biological activity of FGF-2 released from the multifunctional hydrogel following repeated NIR irradiation was evaluated using Cell Counting Kit-8 (CCK-8) and scratch wound healing assays. The Gel@MoO₂ + CIP + FGF-2 hydrogels were incubated in PBS (pH 7.4) at 37 °C, with or without repeated NIR irradiation (808 nm, 0.75 W cm^−2^, 5 min per cycle), to obtain the release media. The concentration of released FGF-2 was quantified by ELISA, and the release media was diluted with complete culture medium to obtain an equivalent FGF-2 concentration before cell treatment. L929 fibroblasts were seeded in 96-well plates at 5 × 10^3^ cells/well and incubated overnight for the CCK-8 assay. The cells were then treated with culture medium (control), gel release medium, free FGF-2, release medium from non-irradiated hydrogel [released FGF-2 (NIR−)], or release medium from NIR-irradiated hydrogel [released FGF-2 (NIR+)]. Cell viability was measured at 24, 48, and 72 h by adding 10 μL of CCK-8 reagent (Dojindo Laboratories, Japan) to each well and incubating for 2 h at 37 °C. The absorbance was measured with a microplate reader (BioTek Instruments, USA) at 450 nm. For the scratch wound healing assay, L929 fibroblasts were seeded in 6-well plates and cultured until they were approximately 90% confluence. A uniform scratch was made with a sterile 200 μL pipette tip, and detached cells were removed by washing with PBS. The cells were then treated with culture medium (control), gel release medium, free FGF-2, released FGF-2 (NIR−), or released FGF-2 (NIR+). Images of the scratch area were taken at 0 and 24 h by an inverted optical microscope (Olympus, Japan). The wound closure percentage was quantified using ImageJ software (NIH, USA) according to the equation provided in Section 2.7.3. All the experiments were performed in triplicate, and the results are presented as mean ± standard deviation (SD).

### 2.8. *In vivo* wound healing studies

#### 2.8.1. Animal model establishment

All the animal experiments were conducted between June 10 2025 and September 15 2025, at the Chinese People's Liberation Army (PLA) Hospital Research Animal Facility. All procedures were carried out in accordance with the ARRIVE 2.0 guidelines and were approved by the Institutional Animal Care and Use Committee (IACUC) of the Chinese People's Liberation Army (PLA) Hospital under Animal Ethics Approval Number: S2025-145-03. All experimental procedures complied with national animal welfare regulations and internationally accepted veterinary guidelines, including the American Veterinary Medical Association (AVMA) Guidelines for the Euthanasia of Animals (2020). The institutional Animal Licence Number was SYXK (Beijing) 2023-0007.

Male Sprague-Dawley rats (200–250 g) were housed in separate ventilated cages under standard laboratory conditions (temperature 22 °C ± 2 °C, relative humidity 50%–60%, 12 h light/dark cycle) with free access to food and water. Before the experiment, the animals were adapted to the laboratory conditions for one week to minimize stress-related variations. Animals were randomly assigned to five experimental groups (*n* = 5 per group) using computer-generated randomization to reduce selection bias. The investigators performing the histopathological evaluation were blinded to the treatment allocation during the analysis in order to reduce observational bias. Histological sections were coded before examination, and group identities were not revealed until all assessments and data recordings were completed.

To establish a full-thickness excision wound model, the rats were anesthetized with an intraperitoneal injection of xylazine (10 mg/kg) and ketamine (80 mg/kg). Adequate depth of anesthesia was confirmed by the absence of the pedal withdrawal reflex and by regular respiratory rate monitoring. The dorsal fur was shaved, and the exposed area was disinfected with 70% ethanol. Using a sterile biopsy punch, a circular full-thickness wound (diameter 10 mm) was made on the mid-dorsal region of each rat, avoiding the spinal area. Hemostasis was achieved by gentle blotting with sterile gauze. The next day, 100 µL of *Staphylococcus aureus* suspension (1 × 10^8^ CFU/mL) was topically applied to each wound to mimic an infected wound model. The animals were then housed in a sterile environment for 24 h to allow the growth of bacterial infection, as evidenced by erythema and exudate formation. After 24 h, the wounds of the control group rats were dressed with gauze; the other group rats were dressed with the respective hydrogel composites and drugs. The hydrogel solution (100 µL) underwent gelation at body temperature, and NIR irradiation (0.75 W cm^−2^ for 7 min) was applied to the hydrogel for 7 min. Medical tape was used to cover the hydrogel dressing from being chewed by the rats. Photographs of the dorsal wounds were taken at the specified periods. The wound healing ratio was measured using the following equation:

$$\text{Relative wound area}\;(\%)=\frac{S_{c}}{S_{i}}\times 100,$$

where S_i_ and S_c_ represent the size of the initial and current wound, respectively. Body weight and signs of infection were recorded every day throughout the experiment to monitor treatment tolerance and systemic health.

#### 2.8.2. Histopathological studies

At the end of the experimental period (day 11), all the animals were euthanized to obtain tissues. Euthanasia was performed using an overdose of pentobarbital sodium (150 mg/kg, i.p.). The procedure was in accordance with the AVMA Guidelines for the Euthanasia of Animals (2020). Death was determined by cessation of heartbeat and respiration. The full-thickness skin with the regenerated wound area and adjacent normal tissue was removed, immediately fixed in 10% neutral buffered formalin for 24 h, then processed for paraffin embedding, dehydrated in graded ethanol, sectioned at 5 μm with a rotary microtome, and stained with hematoxylin and eosin (H&E) for microscopic examination. Sections were stained with H&E to assess epithelial regeneration, granulation tissue formation, and inflammatory cell infiltration. The stained slides were examined under a light microscope (Leica DM5000B, Germany) for epithelial thickness, fibroblast density, and collagen organization. A blinded histopathologist performed semi-quantitative scoring of healing parameters, including inflammation, re-epithelialization, angiogenesis, and collagen maturation.

#### 2.8.3. Biochemical parameters

For biochemical analysis, wound tissue homogenates were prepared by mixing 100 mg of freshly excised wound tissue with 1 mL of ice-cold PBS (pH 7.4) and centrifuging at 1000 rpm for 10 min at 4 °C. The supernatant was collected to test oxidative stress markers. The thiobarbituric acid reactive substances (TBARS) method was used to study lipid peroxidation by measuring the levels of malondialdehyde (MDA), which are expressed as nmol of MDA per mg of protein. Commercially available assay kits (Sigma-Aldrich) were used to quantify the antioxidant enzyme activities, including superoxide dismutase (SOD) and catalase (CAT), according to the manufacturer's instructions. Higher levels of SOD and CAT and lower levels of MDA indicate reduced oxidative stress and improved redox balance at the wound site.

#### 2.8.4. Quantitative real-time PCR analysis

To understand the molecular mechanism behind wound healing and tissue regeneration, total RNA was isolated from wound tissues using TRIzol reagent (Invitrogen, USA). A NanoDrop spectrophotometer (A260/A280 = 1.8–2.0) was used to assess RNA purity and integrity. Then, 1 µg of RNA was reverse transcribed into complementary DNA (cDNA) using a high-capacity cDNA synthesis kit from Takara, Japan. We used a StepOnePlus Real-Time PCR system and SYBR Green Master Mix (Applied Biosystems, USA) to perform quantitative real-time PCR (qRT-PCR). Gene-specific primers targeted inflammatory markers (IL-6, TNF-α), angiogenic genes (FGF-2, VEGF), extracellular matrix remodeling markers (MMP-9, Col-1), and oxidative stress–related genes involved in the Nrf2/Keap1 signaling pathway (Nrf2, Keap1, and HO-1). β-actin was used as the housekeeping gene. The primer sequences are provided in Table SI 2. The 2^−^^ΔΔCt^ method was used to calculate the relative gene expression levels. We evaluated the fold changes in expression across groups to examine how the composite hydrogel influences inflammation, angiogenesis, and collagen remodeling.

### 2.9. Statistical analysis

All experiments were performed in triplicate (*n* = 5 per group for *in vivo* studies). The data are expressed as mean ± SD. Statistical significance was determined using one-way ANOVA followed by Tukey's post hoc test (*p* < 0.05 considered statistically significant).

## 3. Results and discussion

### 3.1. Physicochemical characterization

**Scheme 1.**
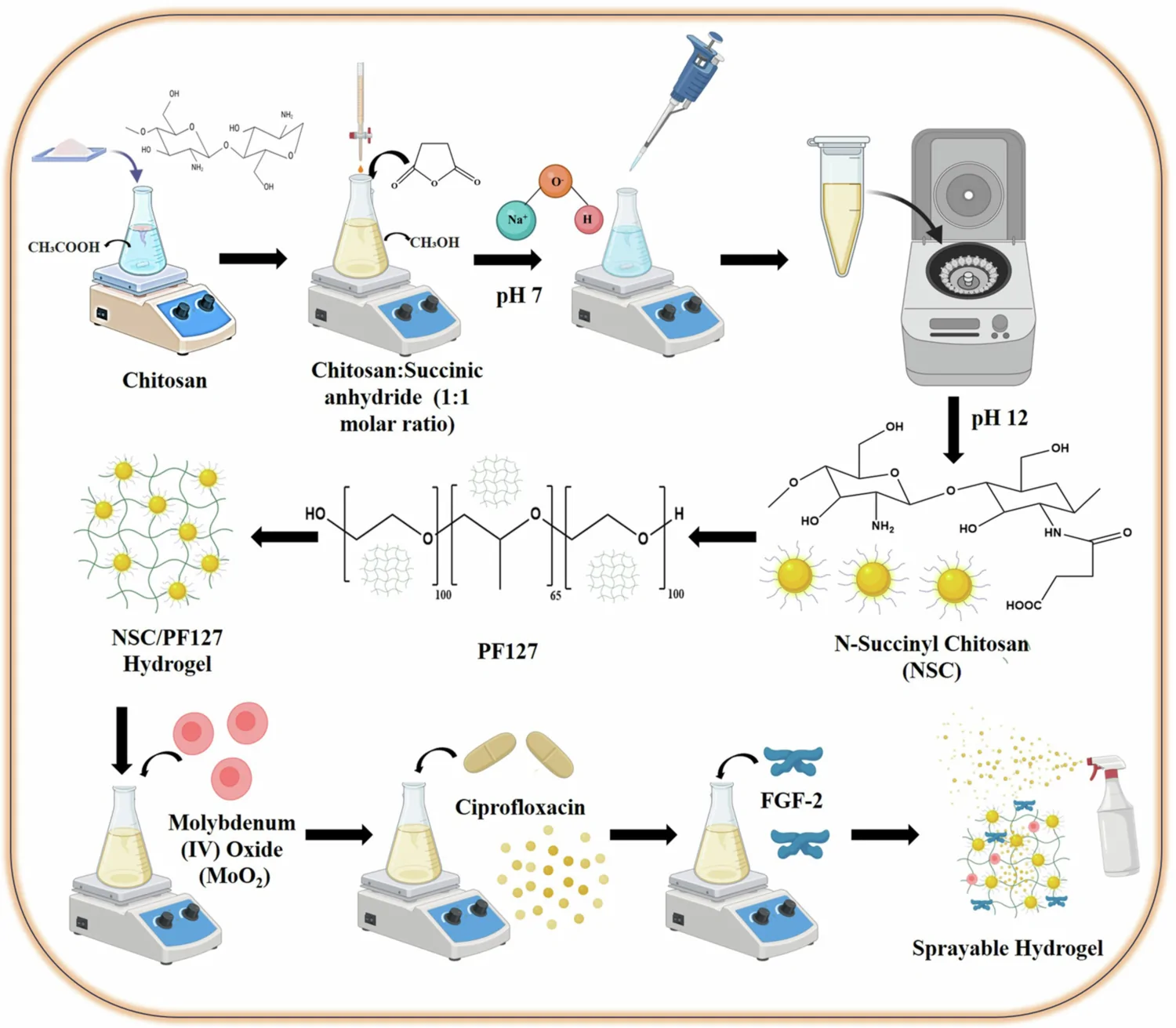
Schematic representation of the synthesis of NSC, NSC/PF127 sprayable hydrogel, and the incorporation of MoO_2_ nanoparticles, CIP, and FGF-2 on sprayable hydrogel (Gel@MoO_2_ + CIP + FGF-2).

**Figure 1.**
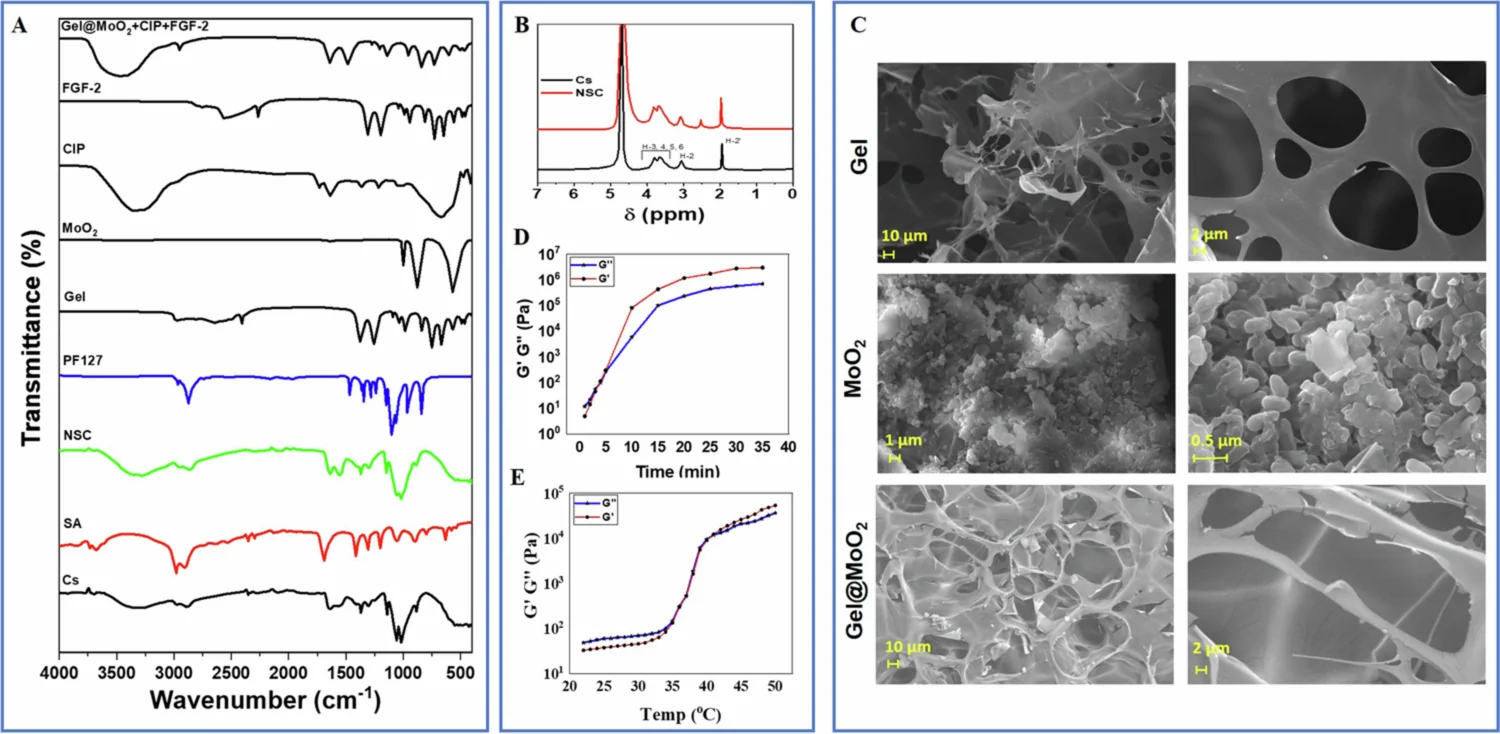
(A) FT-IR spectra of CS, SA, NSC, PF127, gel (hydrogel), MoO_2_, CIP, FGF-2, and Gel@MoO_2_ + CIP + FGF-2. (B) ^1^H-NMR spectra of chitosan (CS) and N-succinyl chitosan (NSC) products. (C) SEM images of the hydrogel, MoO_2_, and Gel@MoO_2_ composites. (D) Storage and loss modulus of NSC/PF127 hydrogel as a function of time (1—35 min). (E) Storage and loss modulus of NSC/PF127 hydrogel as a function of temperature (22 °C–50 °C).

The detailed synthesis of NSC, the NSC/PF127 sprayable hydrogel, and the incorporation of MoO_2_ nanoparticles, CIP, and FGF-2 on the sprayable hydrogel (Gel@MoO_2_ + CIP + FGF-2) are depicted in Scheme 1. The FT-IR spectra of CS, SA, and the formed NSC were first analyzed to confirm the successful formation of NSC, as shown in Figure 1A. CS exhibited its characteristic peaks at ~3400 cm^−1^, which are responsible for the O–H/N–H stretching band, and amide I and II bands were observed at ~1650 cm^−1^ and ~1580 cm^−1^, respectively. Saccharide skeletal vibrations were observed in the region of 1150–900 cm^−1^. SA exhibited strong C = O stretching peaks of the anhydride group at ~1840 and ~1780 cm^−1^ in addition to C–O stretching vibrations at ~1040–950 cm^−1^ (Bashir et al. 2019). After the reaction, the NSC showed clear and distinct changes in the peaks of the starting materials: (i) the absence of the anhydride C = O stretching peaks of SA, (ii) the emergence of a new, broadened amide I band near ~1715–1725 cm^−1^, and (iii) an intensified amide II band at ~1550–1570 cm^−1^. These changes confirmed the ring opening of SA and the formation of amide linkages with the amino groups of CS. The presence of stable polysaccharide skeletal vibrations indicates successful N-succinylation without disturbing the chitosan backbone (Fan et al. 2023). The FT-IR spectrum of Pluronic F127 (PF127) was dominated by the C–H stretching vibrations of the poly(ethylene oxide)/poly(propylene oxide) blocks at ~2880–2950 cm^−1^ and the strong ether C–O–C stretch around ~1100 cm^−1^. The FT-IR spectrum of the hydrogel prepared using the synthesized NSC and PF127 exhibited all the expected peaks of both materials, but with clear shifts in peak positions and intensities associated with intermolecular interactions. In particular, the O–H/N–H stretching band was further widened and slightly shifted, indicating the formation of a hydrogen bond between NSC and PF127 chains. In addition, peak merging and the intensity reductions in the NSC amide and PF127 ether peaks suggest that supramolecular stabilization is consistent with gel assembly. These spectral characteristics confirm the successful formation of the hydrogel (Rungrod et al. 2025; Patel et al. 2022). MoO_2_ nanoparticles exhibited metal‒oxygen vibrational bands, primarily in the 900–600 cm^−1^ region responsible for Mo = O stretching. After encapsulation within the hydrogel matrix, these vibrational bands remained present, albeit with slightly reduced intensity, indicating physical interactions rather than chemical bonding. CIP displayed its fingerprint bands, including quinolone C = O stretching at ~1620–1680 cm^−1^, a C–F band near ~1280–1320 cm^−1^, and aromatic ring vibrations in the ~1500–1450 cm^−1^ region. These bands were retained in the composite spectrum (Gel@MoO₂ + CIP + FGF-2) but appeared widened and slightly shifted, which was consistent with drug‒polymer interactions through hydrogen bonding or electrostatic interactions. The spectrum of FGF-2 exhibited amide I and II bands typical of proteinaceous materials (~1650 cm^−1^ and ~1540 cm^−1^), and the corresponding features were retained in the composite, suggesting successful incorporation of the growth factor into the hydrogel matrix. The composite spectrum thus contains identifiable contributions from NSC, PF127, MoO₂, CIP, and FGF-2 without introducing any new or unexpected peaks. The presence of all the characteristic absorptions, together with slight shifts and broadening in hydrogen-bond-sensitive regions, indicates that the integration of nanoparticles, drug molecules, and protein occurred primarily through physical interactions rather than chemical bonding (Bavarsad et al. 2023; Zheng et al. 2023).

The structural conversion of CS to NSC was further confirmed using ^1^H-NMR spectroscopy (Figure 1B). The spectrum of CS exhibited its characteristic proton resonances, including the methyl protons of the residual N-acetyl groups at ~1.96 ppm, the H-2 signal of the glucosamine unit at ~3.05–3.10 ppm, and a series of overlapping signals between ~3.55 and 3.85 ppm corresponding to H-3, H-4, H-5, and H-6 of the polysaccharide backbone. These resonances are consistent with the characteristic spectral features of partially deacetylated chitosan and serve as an internal reference for assessing further chemical modifications. The spectra of the synthesized NSC revealed several notable alterations after the reaction with succinic anhydride, confirming effective N-succinylation. Significantly, a new set of methylene proton signals that were caused by the succinyl moiety's –CH₂–CH₂– groups emerged in the ~2.25–2.65 ppm range. These peaks were absent from the native CS spectra, and their distinct appearance indicates that succinyl groups were introduced via amide bonds. Similar to chitosan derivatives with additional functional groups, the original polysaccharide backbone signals were still intact but showed a modest widening (Suryani et al. 2024). In accordance with the decreased electron density upon substitution at the amino site, the glucosamine unit's H-2 resonance also showed a small downfield shift relative to that of CS. The simultaneous presence of both characteristic CS protons and newly formed succinyl methylene resonances indicated that the backbone structure remained the same while the modification was made. The spectral pattern of NSC closely matches the characteristic peaks of NSC derivatives presented in the literature (Bavarsad et al. 2023).

Scanning electron microscopy (SEM) was used to examine the morphology of the gel, MoO₂ nanoparticles, and Gel@MoO_2_ (Figure 1C). The pristine gel exhibited a well-defined, continuous three-dimensional network, confirming successful gelation and structural integrity. SEM images of the MoO₂ nanoparticles revealed an irregular morphology with an average particle size of approximately 220 nm, which is consistent with nanoscale oxide particles and moderate aggregation due to high surface energy (Liang et al. 2020). In Gel@MoO_2_, the characteristic hydrogel matrix was preserved, with nanoparticles clearly embedded and distributed throughout the polymer network without disrupting its architecture. The nanoparticles were observed within the hydrogel pores and along the matrix walls, indicating effective loading and good compatibility between the inorganic and polymeric phases. These morphological features collectively confirm successful hydrogel formation and efficient incorporation of MoO₂ nanoparticles into the hydrogel matrix, providing a structural foundation for its potential functional applications (Huang et al. 2022).

### 3.2. Mechanical, swelling, and release characteristics of the NSC/PF127 hydrogel

Although the gel is used in a hydrated state in clinical settings, drying was performed solely for carrying out standard physicochemical studies, such as swelling capacity, degradation, and morphology. The thermoresponsive behavior of the NSC/PF127 hydrogel was evaluated using oscillatory rheology (Figure 1D). At temperatures below 32 °C, the hydrogel displayed low moduli (G′ and G″ ≈ 10–80 Pa), with G″ slightly higher than G′, indicating that the viscous solution is suitable for injectable delivery. As the temperature gradually increased to body temperature, a sharp increase in both moduli occurred between 34 and 38 °C, during which G′ surpassed G″, marking the sol–gel transition. This rapid increase (approximately three orders of magnitude) is attributable to PF127's ability to form micelles and networks as the temperature changes. At temperatures greater than 40 °C, the hydrogel reached a stable elastic plateau (G′ ≈ 10^4^–10^5^ Pa), showing the formation of a strong, physically crosslinked structure. The proximity of the gelation points to 37 °C confirms the material's suitability for *in situ* gelation under physiological conditions, supporting its use in biomedical applications requiring localized stabilization and controlled release. Time-dependent rheological analysis confirmed that the NSC/PF127 composite gels rapidly formed (Figure 1E). Upon completion of the gelation conditions, both G′ and G″ increased sharply, shifting from values close to fluid (<10 Pa) to several orders of magnitude in the first 10–15 min. When G′ exceeded G″, the system transitioned from a viscous solution to an elastic network. After 20 min, the moduli started to stabilize (G′ and G″ in the 10^5^–10^6^ Pa range), indicating that a stable, physically crosslinked gel was formed. The rapid initial increase in *G*ʹ followed by its dominance over *G*" is in line with the thermally driven micellization and network assembly observed in the temperature sweep. This finding shows that the hydrogel not only gels at physiological temperature but also hardens sufficiently for practical biomedical use. This time-resolved profile indicates that the system is suitable for *in situ* gelation applications where predictable and effective mechanical stabilization is crucial (Zhang et al. 2023; Pereira Parchen et al. 2025).

The swelling behavior of the NSC/PF127 hydrogel and its composite formulations was evaluated over 24 h to assess the impact of MoO₂ nanoparticles, CIP, FGF-2, and NIR irradiation on water uptake (Figure SI 1A). The base hydrogel sample demonstrated a rapid initial swelling. Within the first few hours, it reached equilibrium and stabilized at approximately 40–45%. This swelling profile reflects the hydrophilic nature of the NSC backbone and the micellar architecture of PF127, which collectively promote efficient fluid absorption while maintaining structural integrity. The incorporation of MoO₂ or CIP slightly reduced swelling relative to that of the native gel. This change suggests that the added components may limit network expansion by increasing the matrix density or introducing additional physical interactions within the polymeric network. Similarly, the gel containing FGF-2 showed a small decrease in swelling, likely due to protein–polymer interactions that subtly tighten the hydrogel network (Liu et al. 2020). The Gel@MoO₂ + CIP + FGF-2 showed a noticeable reduction in swelling compared to other formulations. The observation could be attributed to the combined effects of all the incorporated agents, which occupy the available free spaces within the polymer network and strengthen internal packing (Shahi et al. 2017). Further, the composite displayed a minor difference in swelling between the NIR– and NIR+ conditions. Although MoO₂ has photothermal activity, the gentle irradiation used here did not alter the polymer structure or its hydration pattern, allowing the gel to retain its physicochemical stability under both conditions. The stable swelling behaviour indicates that the incorporation of MoO₂, CIP, and FGF-2 does not disrupt the hydrogel network, which is advantageous for maintaining moisture balance and sustained therapeutic release.

The ability to hold water by the NSC/PF127 hydrogel was measured to check the stability of the polymer network after swelling. The gel exhibited a slow release of mass, retaining about one-third of its mass after 400 h. This kind of performance is attributed to the physical cross-linking nature of the polymer network, whereby water can be held without damaging the polymer structure (Liu et al. 2023). The addition of MoO₂, CIP, and/or FGF-2 had no notable effect on the above pattern, showing that these compounds can easily be incorporated into the matrix without compromising its structure (Chen et al. 2023). The Gel@MoO₂ + CIP + FGF-2, under both NIR treatment conditions, exhibited a comparable degradation pattern, but with a relatively greater mass loss as a result of increased chain flexibility.

Under mildly acidic conditions (pH 5.5; Figure SI 1C), the hydrogels exhibited a relatively faster mass loss compared to pH 7.4. This behavior can be attributed to increased protonation of amino groups in the NSC matrix, which weakens intermolecular interactions and promotes network relaxation and enhanced water uptake. However, all the systems exhibited a well-controlled degradation pattern, with more than 25% remaining from their original mass after 400 h. The incorporation of MoO₂, CIP, and FGF-2, together with NIR, did not show a significant effect on the degradation performance of the system, except for a minor mass loss due to increased chain mobility in response to NIR. The higher degradation rate at pH 5.5 may be due to the protonation of amino groups in the NSC matrix, which destabilizes the intermolecular bonds that contribute to the relaxation of the network. All formulations were, however, able to achieve controlled rates of degradation, demonstrating adequate stability at physiological and slightly acidic pHs.

Release profiles of CIP from Gel@MoO₂ + CIP + FGF-2 were obtained at two different pH values (7.4 and 5.5) to analyze the impact of surrounding conditions on drug release behavior (Figure SI 1D). In both cases, a rapid release rate was initially observed within the first few hours, after which a sustained release phase was observed. The amount of drug released was greater at pH 7.4 (around 80%–85%) than at pH 5.5 (around 60%–65%). This can be attributed to the higher solubility of CIP under physiological conditions, which facilitates its diffusion through the gel matrix (Ghaffari et al. 2023). However, under acidic conditions, lower solubility and increased binding of the protonated polymer matrix with CIP lead to decreased drug mobility, thus affecting release rates (Akram et al. 2023). This highlights the ability of the hydrogel to provide an initial burst release of the drug along with sustained antibacterial effects.

The release behavior of FGF-2 (Figure SI 1E) showed a biphasic trend, where the first phase consisted of rapid release, followed by sustained delivery. In comparison with CIP, there was an enhanced release at pH 7.4 (about 80–85 ng/mL) than at pH 5.5 (about 70–75 ng/mL) after 72 h. This is attributed to enhanced protein diffusion at lower proton concentrations, while at high proton concentrations, strong interactions take place inside the matrix, retaining FGF-2 molecules. Similar pH-dependent release profiles of CIP and FGF-2 indicate that diffusion through the hydrated hydrogel matrix is the primary release mechanism, an advantage for the coordinated delivery of antibacterial and regenerative agents.

To further elucidate the release mechanism of CIP and FGF-2 from the multifunctional hydrogel, the experimental release data were fitted to five mathematical models, namely, zero-order, first-order, Higuchi, Korsmeyer–Peppas, and Hixson–Crowell models (Figure SI 2 and Table SI 1). Among the other kinetic models, the Korsmeyer–Peppas model showed the best fit for physiological release (pH 7.4) and mildly acidic release (pH 5.5) for both CIP and FGF-2 release, with the highest *R* ^2^ values (0.9434–0.9722), the highest adjusted *R* ^2^ values (0.9245–0.9630), and the lowest RMSE values (0.0226–0.0357).

The corresponding release exponent (*n*) values were 0.4350 and 0.6492 for CIP at pH 5.5 and pH 7.4, respectively, and 0.5139 and 0.4333 for FGF-2 at pH 5.5 and pH 7.4, respectively. The *n* values of approximately 0.43 indicate that diffusion through the hydrated polymer matrix was the predominant release mechanism, whereas values between 0.45 and 0.89 suggest anomalous (non-Fickian) transport involving the combined contribution of diffusion and polymer relaxation. These results suggest that the controlled release behavior of the hydrogel is controlled mainly by diffusion, with a partial contribution of polymer relaxation depending on the encapsulated therapeutic agent and release environment. This release mechanism is in agreement with the swelling and gradual degradation behavior of the hydrogel, providing an initial burst release with sustained therapeutic delivery for a long time.

The Gel@MoO₂ + CIP + FGF-2 showed a well-defined and reproducible photothermal response for four consecutive NIR on‒off cycles (Figure SI 1F). During each cycle, the temperature increased rapidly from room temperature to nearly 50 °C under irradiation, confirming the efficient light-to-heat conversion of the embedded MoO₂. After terminating the NIR exposure, the composite returned to the baseline temperature, and this heating–cooling profile remained unchanged in all cycles.​ The absence of changes in peak temperature indicates that the MoO_2_ remains well dispersed and structurally intact within the hydrogel network during repeated NIR irradiation cycles (Wang et al. 2022). Such reliable cycling behavior is advantageous for applications requiring quite controlled thermal pulses, including sustainable drug release and antimicrobial activation (Zhang et al. 2023). Overall, the composite exhibited robust, reversible photothermal performance that was compatible with the hydrogel's previously demonstrated structural integrity and hydration stability. The injectability of the hydrogel was evaluated qualitatively by extruding the material through needles of different gauges (16, 18, 20, 24, 26, and 31 G). As shown in Figure SI 1G, the hydrogel can be extruded smoothly without phase separation or clogging, demonstrating good flowability and handling properties. Together with the observed sol–gel transition behavior and rheological properties, these results support the suitability of the hydrogel for injectable and minimally invasive wound applications.

### 3.3. *In vitro* antibacterial evaluation of hydrogel formulations

Multiple complementary assays were used to characterize the antibacterial performance of the hydrogel formulations. MIC analysis was used to determine the concentration-dependent inhibition of planktonic bacteria, CFU enumeration was used to quantify surviving viable bacteria, an agar diffusion assay was used to assess diffusion-dependent antibacterial activity, and crystal violet staining was used to evaluate the inhibition of established biofilms. Accordingly, each assay is discussed with emphasis on its specific biological information rather than repeating the same treatment-related trends.

#### 3.3.1. MIC analysis against gram-negative and gram-positive bacteria

The antibacterial efficacy of CIP, hydrogel-based formulations, and Gel@MoO₂ + CIP + FGF-2 composites were investigated through MIC assays against *E. coli*, *P. aeruginosa*, and *MRSA*(Figure SI 3). Free CIP exhibited strong antibacterial activity against the Gram-negative strains, with a mean MIC of 0.25 µg/mL (*n* = 3) for both *E. coli* and *P. aeruginosa*, whereas a higher MIC of 4 µg/mL was observed for MRSA, which is consistent with its reduced susceptibility profile (Figure SI 2B) (Almuhayawi et al. 2023). The Gel did not show measurable antibacterial activity against any of the tested organisms, with MIC values exceeding 100 µg/mL, confirming that the polymeric matrix does not intrinsically inhibit bacterial growth (Figure SI 3A). The gel + CIP formulations retained antibacterial activity, although a moderate increase in MIC values was observed relative to those of the free drug (Figure SI 3C). Specifically, MICs of 0.5 µg/mL were recorded for *E. coli* and *P. aeruginosa*, while MRSA exhibited an MIC of 8 µg/mL. This shift likely reflects the controlled release and diffusion effects associated with drug encapsulation rather than the loss of antimicrobial function (Ghaffari et al. 2023). In contrast, Gel@MoO_2_ without antibiotic loading did not achieve defined MIC values within the tested concentration range (Figure SI 4A). Neither *E. coli* nor *P. aeruginosa* showed growth inhibition up to 32 µg/mL, and MRSA was unaffected at 100 µg/mL. Gel@MoO_2_ with NIR irradiation inhibited the growth of *E. coli* and *P. aeruginosa* at approximately 90 µg/mL but did not show any measurable MIC against MRSA (Figure SI 4B). The results demonstrate that MoO_2_ alone shows limited antibacterial activity under the tested conditions and NIR activation leads to an observable antibacterial response, predominantly against the Gram-negative strains. When CIP was loaded into the MoO_2_-containing hydrogel (Gel@MoO_2_ + CIP + FGF-2), the MIC values of *E. coli* and *P. aeruginosa* were 0.5 μg/mL and 4 μg/mL for MRSA, respectively, comparable to or lower than those of the CIP-loaded hydrogel without nanoparticles (Figure SI 5A). Under NIR irradiation, the MIC of the composite decreased to 0.25 µg/mL for both E. coli and *P*. aeruginosa, similar to that of free CIP, and was still 4 µg/mL for MRSA (Figure SI 5B) (Li et al. 2024). Overall, the MIC data confirmed the concentration-dependent antibacterial activity of the formulations and showed that NIR activation of the MoO_2_-containing composite increased planktonic growth inhibition, particularly against the Gram-negative strains. The higher MIC for MRSA indicates comparatively lower susceptibility under the conditions tested.

#### 3.3.2. Agar well diffusion assay for antibacterial activity evaluation

The antibacterial activity of the synthesized composite was evaluated along with CIP against *E. coli, P. aeruginosa,* and MRSA using the agar well diffusion method. Figure SI 6A presents the representative digital photographs of the inhibition zones, while Figure SI 6B summarises the corresponding zone diameters. The control (distilled water) showed no visible inhibition against any of the tested strains, confirming normal bacterial growth and validating the assay conditions. NIR irradiation alone also failed to produce measurable zones, indicating that NIR exposure in the absence of active agents did not influence bacterial viability (Moorcroft et al. 2020). CIP produced significant zones of inhibition against all three strains, which were about 22.8 ± 0.5 mm against *E. coli,* 24.1 ± 0.6 mm against *P. aeruginosa,* and 24.3 ± 0.9 mm against *MRSA*. Gel + CIP had larger inhibition zones than free CIP against *E. coli, P. aeruginosa,* and *MRSA*, numerically. This suggests that the incorporation of CIP into the hydrogel preserved its diffusion-dependent antibacterial activity (McCoy et al. 2022). The antibacterial activity of the gel alone was very low, with zones less than 10 mm, while the Gel@MoO_2_ showed only a small inhibition, with zones of 21.7 ± 0.6 mm (*E. coli*), 21.8 ± 0.5 mm (*P. aeruginosa*), and 23.7 ± 0.4 mm (*MRSA*). The NIR irradiation of Gel@MoO_2_ increased the respective inhibition zones to 22.9 ± 0.7, 24.2 ± 0.6, and 24.9 ± 0.5 mm, respectively, indicating an NIR-dependent increase in diffusion-based antibacterial activity. The Gel@MoO_2_ + CIP + FGF-2 composite showed measurable zones of inhibition under the NIR− and NIR+ conditions. Without NIR irradiation, the inhibition zones of 21.7 ± 1.2, 21.8 ± 1.1, and 23.7 ± 1.0 mm were observed against *E. coli*, *P. aeruginosa*, and *MRSA*, respectively. The corresponding zones were 22.9 ± 1.3, 24.2 ± 1.2, and 24.9 ± 1.1 mm after NIR irradiation (Zhang et al. 2023; Xu et al. 2025). These results indicate that the NIR-responsive composite can maintain antibacterial activity without irradiation and has enhanced inhibition with NIR irradiation. Compared to the MIC assay, which mainly determines the concentration to inhibit the growth of planktonic bacteria, the results of the agar diffusion provide complementary evidence of the local, diffusion-dependent antibacterial activity of the formulations.

#### 3.3.3. CFU analysis of bacterial viability

**Figure 2.**
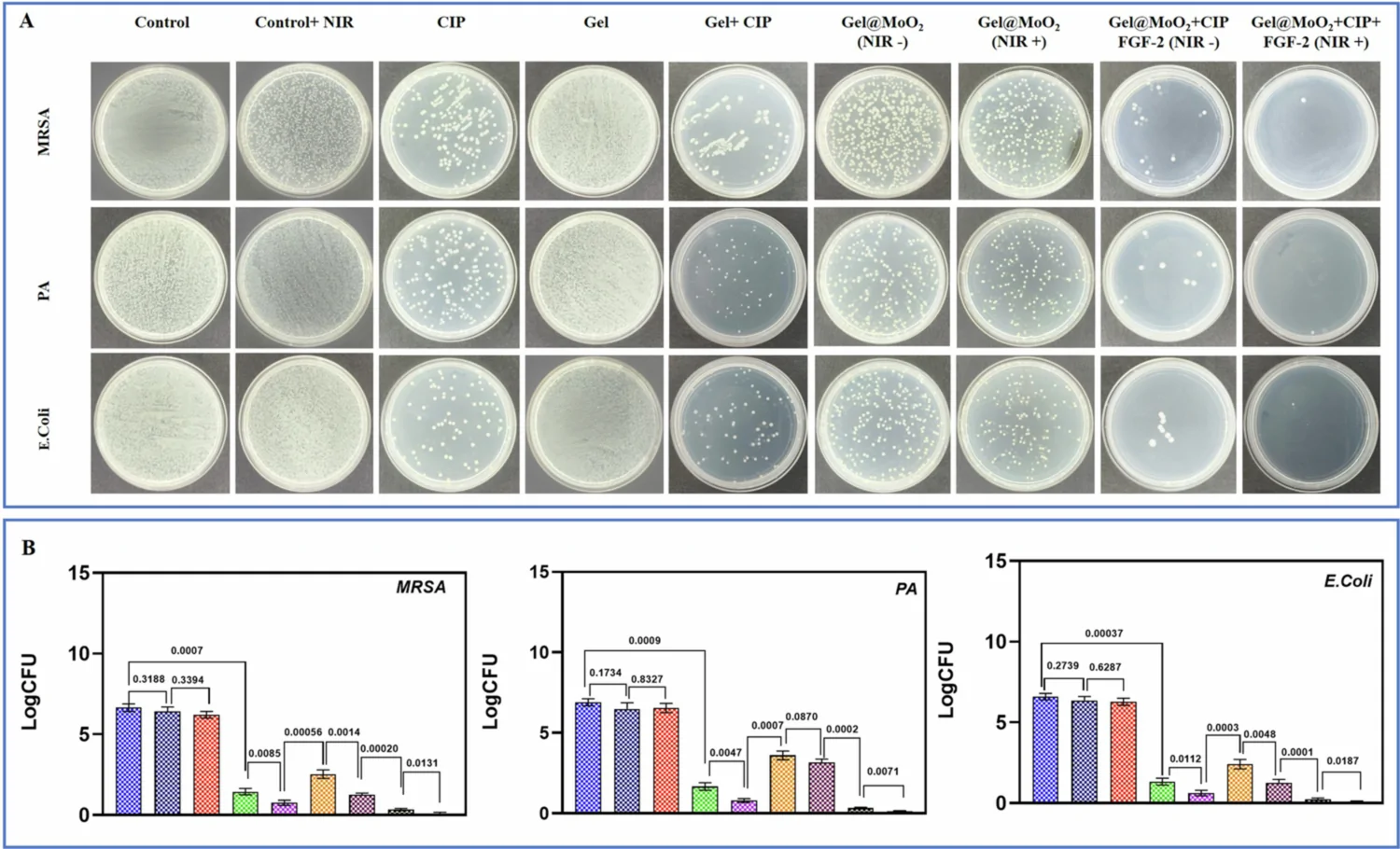
(A) Representative images of the colony formation of the *MRSA, E. coli,* and *P. aeruginosa* incubated with PBS, PBS (irradiated with NIR), CIP (1.25 μg/mL), Gel (100 μg/mL), Gel + CIP (1.25 μg/mL), Gel@MoO_2_ (0.1 mg/mL), Gel@MoO_2_ + NIR (0.1 mg/mL), Gel@MoO_2_ + CIP + FGF-2 (NIR−) (5 mg/mL), and Gel@MoO_2_ + CIP + FGF-2 (5 mg/mL) (NIR+). For the NIR-treated groups, irradiation was performed using an 808 nm laser at 0.75 W cm^−2^ for 5 min. (B) Quantitative data on the colonies were obtained from the agar plates. The data are shown as mean ± SDs (*n* = 3). Statistical analysis was performed using one-way ANOVA followed by Tukey's post hoc test. The exact *p*-values are presented in the figure, and statistical comparisons were performed between the relevant treatment groups, as indicated by the significance bars.

The antibacterial performance of the different formulations was further evaluated by CFU analysis against *E. coli, P. aeruginosa,* and MRSA. Representative agar plate images (Figure 2A) and the corresponding quantitative CFU counts (Figure 2B) are presented to visualize bacterial survival following treatment. The CFU counts demonstrated that the formulations with CIP reduced the viable bacteria, while the blank Gel and the NIR-only groups caused only limited changes in the survival of bacteria. Gel + CIP further decreased viable bacterial counts. The addition of MoO_2_ and NIR irradiation showed more reduction in the composite formulation. The lowest viable bacterial counts were observed in the Gel@MoO₂ + CIP + FGF-2 (NIR+) group, with the reduction being particularly evident for *E. coli* and *P. aeruginosa*. The Gel + CIP formulation showed reduced colony formation, with fewer colonies on the agar plates, compared to the respective untreated control. The CFU counts were slightly changed by NIR irradiation of Gel@MoO_2_. However, the NIR irradiation of Gel@MoO_2_ resulted in a further decrease in the number of viable bacteria, especially for *E. coli* and *P. aeruginosa* (Yang et al. 2025).

The relatively lower response observed for *MRSA* was in agreement with its higher susceptibility threshold in the MIC assay. The Gel@MoO₂ + CIP + FGF-2 composite considerably reduced viable bacterial counts under both NIR− and NIR+ conditions. The residual viable bacteria were further decreased by NIR irradiation, with the maximal reduction for *E. coli* and *P. aeruginosa* and a clear decrease in the survival of *MRSA* as compared to the corresponding non-irradiated formulation. Representative agar plates were consistent with the quantitative CFU measurements and showed markedly fewer residual colonies following treatment with the NIR-activated composite (Mahafdeh et al. 2022). Together, the CFU analyses provide direct quantitative evidence of treatment-induced changes in recoverable viable bacteria. These results indicate the contribution of CIP to bacterial killing and the additional effect of MoO_2_-mediated NIR activation in the composite. The CFU assay gives a direct counting of the surviving bacteria after treatment instead of reiterating the results of MIC and agar diffusion (Norris et al. 2022; 2081; Zhang et al. 2023).

#### 3.3.4. Antibiofilm activity of the synthesized hydrogel composites

**Figure 3.**
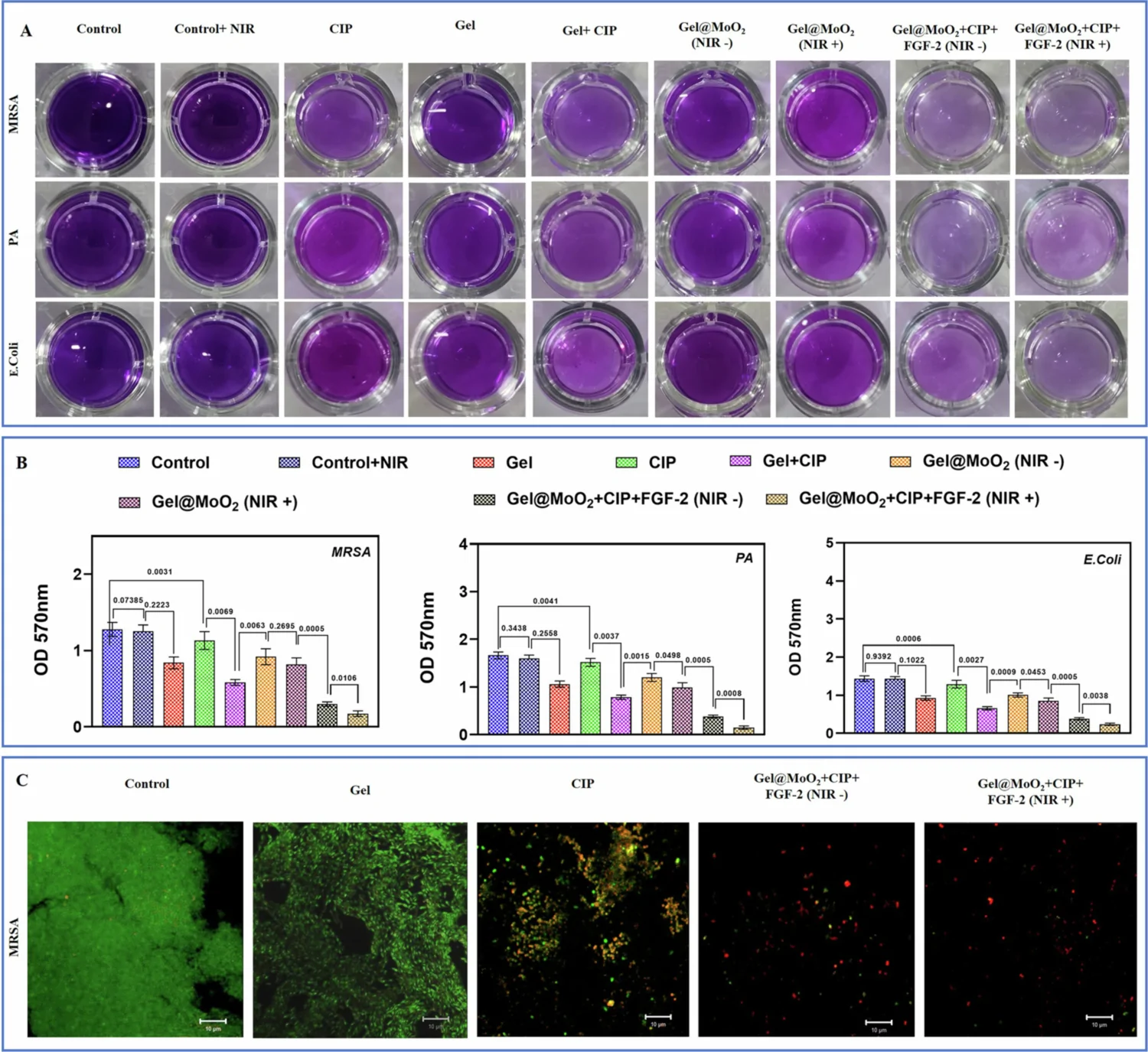
(A) Representative images of the *MRSA, P. aeruginosa, and E. coli* biofilms stained with crystal violet following various therapies. (B) The OD values of the crystal violet staining on *MRSA, P. aeruginosa, and E. coli* biofilms after treatment with PBS, PBS (irradiated with NIR), CIP (1.25 μg/mL), Gel (100 μg/mL), Gel + CIP (1.25 μg/mL), Gel@MoO_2_ (0.1 mg/mL), Gel@MoO_2_ + NIR (0.1 mg/mL), Gel@MoO_2_ + CIP + FGF-2 (NIR-) (5 mg/mL), and Gel@MoO_2_ + CIP + FGF-2 (NIR+) (5 mg/mL). For the NIR-treated groups, irradiation was performed using an 808 nm laser at 0.75 W cm^−2^ for 5 min. The data are presented as the mean ± SD (*n* = 3). Statistical analysis was performed using one-way ANOVA followed by Tukey's post hoc test. Exact *p*-values are presented in the figure, and statistical comparisons were performed between the indicated treatment groups, as denoted by the significance bars. (C) Representative confocal laser scanning microscopy (CLSM) images of AO/EB dual-stained MRSA biofilms after the corresponding treatments, showing viable bacteria (green fluorescence) and membrane-compromised/non-viable bacteria (red fluorescence), thereby confirming the antibiofilm activity of the developed multifunctional hydrogel.

Biofilm formation and inhibition were evaluated using a crystal violet staining assay, with biofilm biomass quantified via optical density measurements at 570 nm (OD₅₇₀) and supported by representative digital photographs (Figure 3A and 3B). The untreated control groups exhibited strong biofilm formation across all three bacterial strains, with OD₅₇₀ values of 1.27 ± 0.05 for *E. coli,* 1.62 ± 0.05 for *P. aeruginosa,* and 1.42 ± 0.05 for MRSA. Exposure to NIR irradiation alone did not significantly alter biofilm biomass, as reflected by comparable OD₅₇₀ values (1.20 ± 0.04 for *E. coli,* 1.55 ± 0.04 for *P. aeruginosa*, and 1.45 ± 0.04 for MRSA), indicating that NIR treatment under the applied conditions does not independently disrupt biofilm development (Hashemi et al. 1925). The Gel group showed only a modest reduction in biofilm biomass relative to the control, with OD₅₇₀ values of 1.10 ± 0.06 for *E. coli*, 1.51 ± 0.06 for *P. aeruginosa*, and 1.25 ± 0.06 for MRSA, indicating limited antibiofilm activity of the hydrogel matrix alone.

Treatment with CIP resulted in a noticeable decrease in biofilm biomass for all strains, yielding OD₅₇₀ values of 0.80 ± 0.03 for both *E. coli* and *P. aeruginosa* and 0.92 ± 0.03 for MRSA. The better reduction observed for the Gram-negative strains was consistent with their comparatively better susceptibility in the planktonic bacterial assays (Banerjee et al. 2023; Chegini et al. 2025). The incorporation of CIP into the hydrogel led to further strain-dependent effects. The Gel + CIP formulation reduced the biofilm biomass to 0.57 ± 0.04 for *E. coli* and 0.65 ± 0.04 for *MRSA*, whereas a higher OD₅₇₀ value of 1.02 ± 0.04 was observed for *P. aeruginosa*, indicating partial but incomplete biofilm suppression. Gel@MoO_2_ nanoparticles without irradiation showed limited antibiofilm activity, with OD₅₇₀ values remaining relatively high (0.96 ± 0.05 for *E. coli,* 1.20 ± 0.05 for *P. aeruginosa*, and 1.03 ± 0.05 for *MRSA*). Upon NIR irradiation, the Gel + MoO₂ group exhibited a moderate reduction in biofilm biomass for all strains, with OD₅₇₀ values decreasing to 0.87 ± 0.04, 0.94 ± 0.04, and 0.84 ± 0.04 for *E. coli, P. aeruginosa*, and *MRSA*, respectively.

The Gel@MoO₂ + CIP + FGF-2 composite demonstrated a substantial reduction in biofilm formation across all bacterial strains, with OD₅₇₀ values of 0.31 ± 0.03 for *E. coli*, 0.37 ± 0.03 for *P. aeruginosa,* and 0.37 ± 0.03 for MRSA under non-irradiated conditions. Following NIR irradiation, the composite produced further reductions in biofilm biomass, with OD₅₇₀ values of 0.17 ± 0.02 for *E. coli*, 0.16 ± 0.02 for *P. aeruginosa*, and 0.23 ± 0.02 for *MRSA*. These quantitative findings were consistent with photographic observations, which showed minimal residual staining and sparse biofilm coverage following treatment with the NIR-activated composite (Ahire et al. 2018). The results extend the antibacterial evaluation to established biofilms by demonstrating a reduction in retained biofilm biomass following treatment. The marked reduction in biofilm biomass observed with Gel@MoO₂ + CIP + FGF-2, particularly after NIR irradiation, indicates enhanced antibiofilm activity under the tested conditions (Ullah et al. 2024).

#### 3.3.5. Qualitative evaluation of bacterial viability within MRSA biofilms by CLSM

To further confirm the antibiofilm activity observed in the crystal violet assay, qualitative analysis of bacterial viability in *MRSA* biofilms after AO/EB dual staining was performed via CLSM (Figure 3C). The untreated control group exhibited a dense and continuous biofilm with green fluorescence, mainly indicating a high proportion of viable bacterial cells and little bacterial death. A similar fluorescence pattern was observed for the Gel group, indicating a limited effect of the blank hydrogel on bacterial viability. Treatment with CIP resulted in a marked reduction in biofilm-associated fluorescence, accompanied by increased red fluorescence. The Gel@MoO_2_ + CIP + FGF-2 (NIR−) group showed only sparse bacterial aggregates with greatly increased red fluorescence and markedly decreased green fluorescence. The Gel@MoO_2_ + CIP + FGF-2 (NIR+) group exhibited the largest antibacterial effect after NIR irradiation, with few residual bacterial cells and mainly red fluorescence, which indicated that most of the bacterial membrane was damaged and only a very small part of the bacteria survived in the biofilm.

### 3.4. *In vitro* biological studies

#### 3.4.1. Cytocompatibility assessments of hydrogel composites

**Figure 4.**
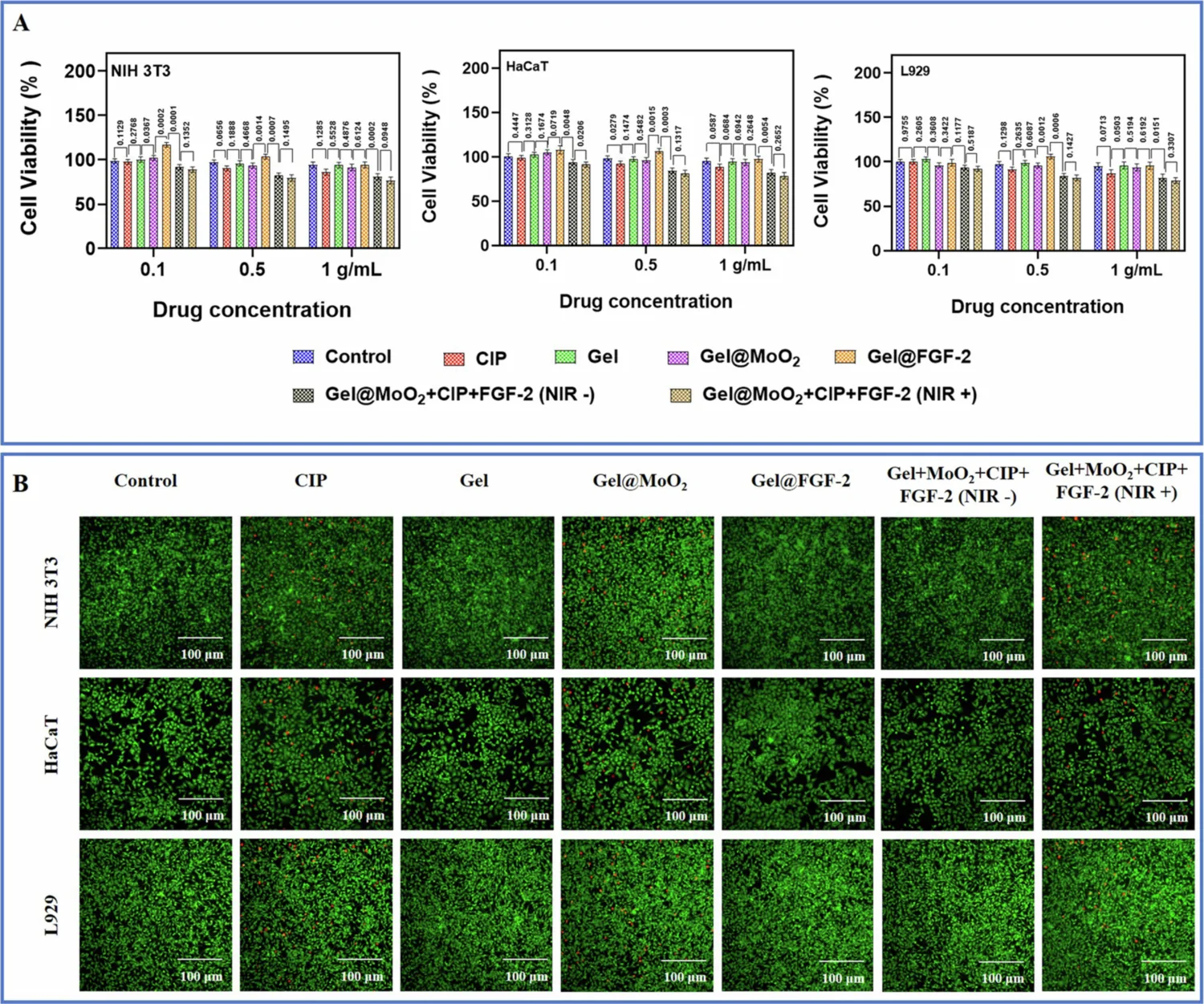
(A) Quantitative cell viability of NIH 3T3, HaCaT, and L929 cells following treatment with PBS, CIP, Gel, Gel@MoO₂, Gel@FGF-2, Gel@MoO₂ + CIP + FGF-2 (NIR−), and Gel@MoO₂ + CIP + FGF-2 (NIR+). (B) Representative fluorescence images of AO/EB-stained NIH 3T3, HaCaT, and L929 cells following the corresponding treatments. For the NIR-treated groups, irradiation was performed using an 808 nm laser at 0.75 W cm^−2^ for 5 min. The data are presented as the mean ± SD (*n* = 3). Statistical analysis was performed using one-way ANOVA followed by Tukey's post hoc test. The exact *p*-values are presented in the figure, and statistical comparisons were performed between the indicated treatment groups, as denoted by the significance bars.

The cytocompatibility of the hydrogel-based composites was evaluated across three different cell lines (L929, NIH3T3, and HaCaT) at increasing concentrations, revealing clear composition and dose-dependent trends (Figure 4A). In all the cases, the control groups maintained a cell viability close to 100%, confirming the suitability of the experimental conditions. Treatment with CIP alone showed a gradual reduction in viability relative to control, but the values remained within an acceptable range (87.03 ± 3.91, 86.13 ± 3.46, and 88.37 ± 3.35 for L929, NIH3T3, and HaCaT, respectively), indicating the absence of severe cytotoxic effects at the analyzed doses. The Gel exhibited excellent biocompatibility (95.92 ± 3.95, 94.03 ± 3.10, and 94.90 ± 3.10 for L929, NIH3T3, and HaCaT, respectively), with viability comparable to or slightly exceeding that of the control, underscoring its suitability as a drug-delivery scaffold (Zhao et al. 2024; Hu et al. 2025). The introduction of MoO₂ resulted in a moderate, concentration-dependent reduction in cell viability across all cell lines, which can be attributed to increased nanoparticle–cell interactions at higher doses, though viability remained above critical toxicity thresholds. Conversely, FGF-2-loaded hydrogels notably increased cell viability, particularly at lower concentrations (95.39 ± 3.99, 94.17 ± 3.40, and 97.37 ± 3.35 for L929, NIH3T3, and HaCaT, respectively), with values surpassing 100% in some cases, reflecting the growth factor's conserved bioactivity and proliferative capacity. The combined Gel@MoO₂ + CIP + FGF-2 formulation under non-irradiated conditions exhibited balanced cytocompatibility (82.12 ± 4.02, 80.93 ± 3.56, and 82.40 ± 3.36 for L929, NIH3T3, and HaCaT, respectively), showing slightly reduced viability compared to FGF-2-only systems but higher viability than the corresponding MoO₂ or CIP-containing formulations under the tested conditions, suggesting a compensatory effect of FGF-2 within the composite (Arslan et al. 2021). Upon NIR irradiation, the Gel@MoO₂ + CIP + FGF-2 group exhibited the most noticeable reduction in cell viability in a dose-dependent manner (78.86 ± 3.15, 76.70 ± 3.83, and 78.70 ± 3.64 for L929, NIH3T3, and HaCaT, respectively), which was consistent with the photothermal activation of MoO₂ and the associated increase in cellular stress; nevertheless, viability at lower and intermediate concentrations remained within a tolerable range. Despite minor variations in absolute viability being observed among the three cell lines, the overall response patterns remained consistent, indicating that the developed hydrogel composites offer a favorable balance between cytocompatibility, growth-promoting efficacy, and externally controllable therapeutic activity, thereby endorsing their potential for sophisticated biomedical and wound-healing applications (Jin et al. 2022; Long et al. 2025).

#### 3.4.2. Live/dead assay by fluorescence (AO/EB) staining

Live/dead fluorescence (AO/EB) staining was used to evaluate the cytocompatibility of the hydrogel composites for L929, NIH3T3, and HaCaT cells, where live cells emit a green color and where dead cells emit a red color. The control groups of all the cell lines showed dense, uniformly distributed green fluorescence with negligible red spots, which indicates normal cell viability (Figure 4B). The cells treated with CIP exhibited some red fluorescence, particularly in NIH3T3 and HaCaT cell lines, which indicated slight cytotoxic stress while maintaining overall cell viability. The Gel sample showed viability comparable to that of the control, with minimal cell death, confirming its intrinsic biocompatibility. The Gel@MoO_2_ showed a modest increase in dead cells, as indicated by red fluorescence, particularly in NIH3T3 and HaCaT cell lines, with prominent green fluorescence, indicating acceptable cytocompatibility. The FGF-2-loaded formulation (Gel@FGF-2) displayed high green fluorescence and high cell density across all the cell lines, confirming its enhanced cell survival and proliferation. The Gel@MoO₂ + CIP + FGF-2 hydrogel formulation in the absence of NIR irradiation maintained high viability with limited red staining, suggesting that FGF-2 mitigated the mild cytotoxic effects of MoO₂ and CIP. Following NIR irradiation, an increase in red fluorescence was noticed, particularly in NIH3T3 and HaCaT cells, which was attributable to photothermal effects; however, live cells remained predominant, especially in the L929 cell line (Wang et al. 2022; Li et al. 2021; Chen et al. 2023). Overall, these observations confirm that the hydrogel composites exhibit good cytocompatibility and a controllable cellular response under NIR activation, which is advantageous for their suitability for biomedical applications.

#### 3.4.3. *In vitro* wound healing assay

**Figure 5.**
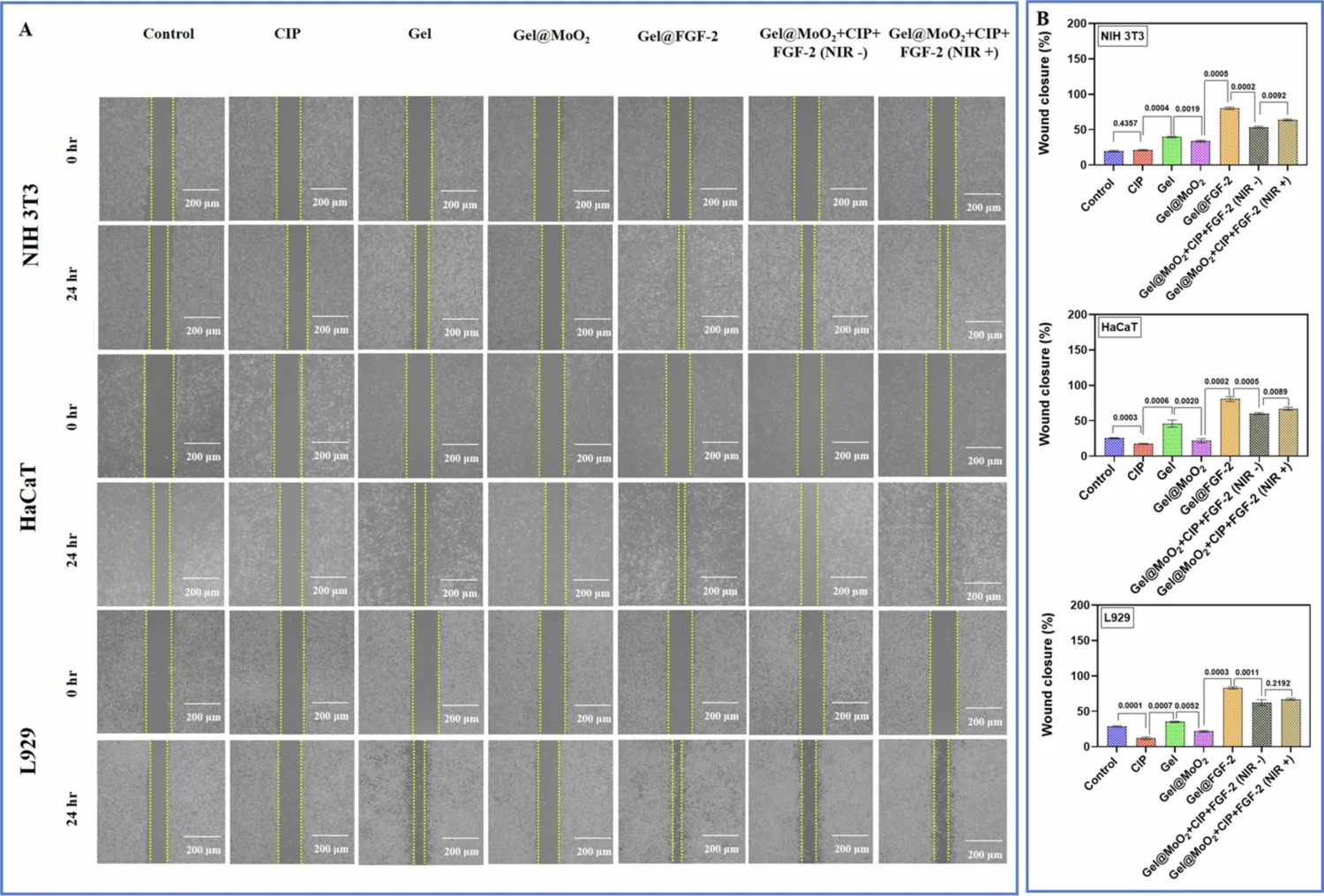
(A) Representative phase-contrast images of NIH 3T3, HaCaT, and L929 cells following treatment with PBS, CIP (1.25 μg/mL), Gel (100 μg/mL), Gel@MoO₂ (0.1 mg/mL), Gel@FGF-2 (0.1 mg/mL), Gel@MoO₂ + CIP + FGF-2 (NIR−) (5 mg/mL), and Gel@MoO₂ + CIP + FGF-2 (NIR+) (5 mg/mL). For the NIR-treated groups, irradiation was performed using an 808 nm laser at 0.75 W cm^−2^ for 5 min. (B) Quantitative analysis of wound closure (%) determined from phase-contrast microscope images of NIH 3T3, HaCaT, and L929 cells. The data are presented as mean ± SD (*n* = 3). Statistical analysis was performed using one-way ANOVA followed by Tukey's post hoc test. The exact *p*-values are presented in the figure, and statistical comparisons were performed between the indicated treatment groups, as denoted by the significance bars.

The wound healing ability of the hydrogel formulations was assessed using an *in vitro* scratch assay on NIH3T3, HaCaT, and L929 cells at 0 and 24 h (Figures 5A and Figure 5B). All groups displayed a uniform scratch width at 0 h, confirming consistent wound generation. After 24 h, the control groups showed partial wound closure (19.63 ± 1.17, 25.50 ± 0.70, and 28.73 ± 0.85 for NIH3T3, HaCaT, and L929 cells, respectively), which was consistent with normal cell migration, whereas CIP-treated cells showed slower closure, mainly in NIH3T3 (21.12 ± 0.91) and HaCaT cell lines (17.30 ± 0.80), indicating a modest inhibitory effect on migration. Gel promoted greater wound closure than the control and CIP groups across the tested cell lines, indicating a supportive microenvironment for cell migration. Gel@MoO_2_ demonstrated moderate migration (33.63 ± 1.25, 21.80 ± 2.95, and 21.63 ± 1.15 for NIH3T3, HaCaT, and L929 cells, respectively), with slightly reduced closure compared to the gel, likely due to nanoparticle-associated cellular stress. In contrast, Gel@FGF-2 exhibited the most pronounced wound closure in all three cell lines (80.17 ± 1.78, 80.53 ± 3.36, and 82.93 ± 1.70 for NIH3T3, HaCaT, and L929 cells, respectively), demonstrating the potent pro-migratory and regenerative effects of FGF-2 on fibroblasts and keratinocytes. This enhanced migration is attributed to FGF-2–mediated stimulation of cytoskeletal remodeling, cell–matrix interactions, and signaling pathways associated with tissue regeneration (Gözde et al. 2024). The Gel@MoO₂ + CIP + FGF-2 composite under non-irradiated conditions also supported effective wound closure (53.37 ± 1.25, 60.13 ± 1.25, and 62.30 ± 4.35 for NIH3T3, HaCaT, and L929 cells, respectively), indicating that FGF-2 mitigates the mild migration-inhibitory effects of CIP and MoO₂. Under NIR irradiation, wound closure was numerically further increased relative to the corresponding non-irradiated composite group. This improvement suggests that controlled photothermal activation may enhance cell migration by promoting localized matrix relaxation and growth factor diffusion without inducing excessive cellular stress. Although complete closure was not achieved within 24 h, the irradiated composite demonstrated a higher migratory response than the corresponding non-irradiated formulation under the tested conditions (Wang et al. 2022). The results indicate that the hydrogel system facilitates cell migration in a composition-dependent manner, with FGF-2 markedly improving wound healing and NIR activation, enabling controllable modulation of the cellular response (Aydın et al. 2024).

#### 3.4.4. *In vitro* cell proliferation assay

**Figure 6.**
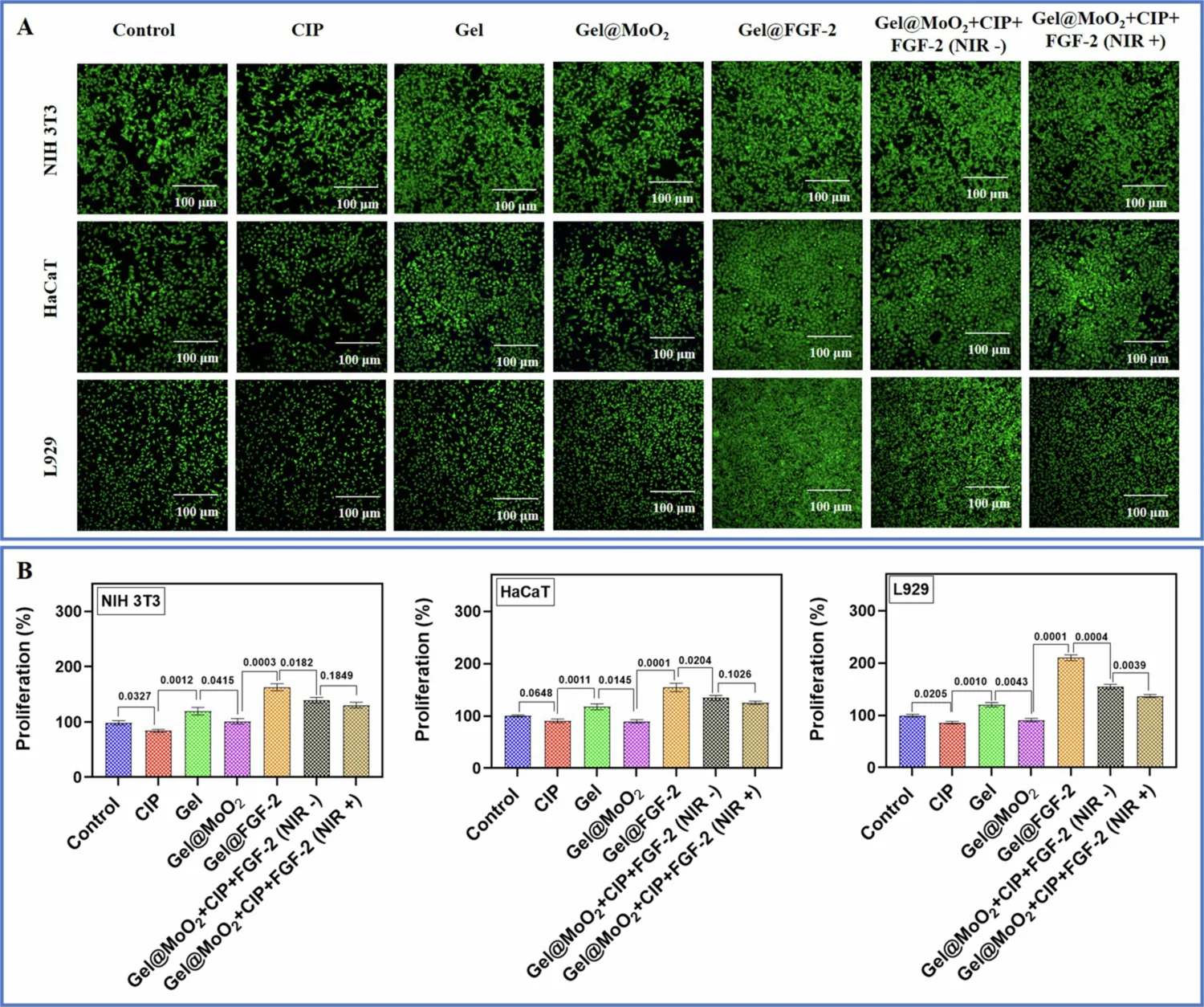
*In vitro* evaluation of the cell proliferation and cytocompatibility of the developed composites. (A) Representative fluorescence images showing the proliferation of NIH 3T3, HaCaT, and L929 cells following treatment with PBS, CIP (1.25 μg/mL), Gel (100 μg/mL), Gel@MoO₂ (0.1 mg/mL), Gel@FGF-2 (0.1 mg/mL), Gel@MoO₂ + CIP + FGF-2 (NIR−) (5 mg/mL), and Gel@MoO₂ + CIP + FGF-2 (NIR+) (5 mg/mL). For the NIR-treated groups, irradiation was performed using an 808 nm laser at 0.75 W cm^−2^ for 5 min. (B) Quantitative analysis of the cell proliferation (%) of NIH 3T3, HaCaT, and L929 cells. The data are presented as mean ± SD (*n* = 3). Statistical analysis was performed using one-way ANOVA followed by Tukey's post hoc test. Exact *p*-values are presented in the figure, and statistical comparisons were performed between the indicated treatment groups, as denoted by the significance bars.

Fluorescence imaging was used to qualitatively evaluate cell proliferation in NIH3T3, HaCaT, and L929 cell lines after being treated with various hydrogel formulations. An increase in cell density and green fluorescence intensity indicates good proliferative activity (Figure 6A and 6B). In all three cell lines, the control groups exhibited a moderate, uniform cell distribution, indicating baseline proliferation under standard culture conditions. The CIP treatment resulted in the lowest cell density across all cell lines, such as ~85% in NIH3T3, ~90% in HaCaT, and ~88% in L929 cells. This indicates that CIP suppresses cellular proliferation despite maintaining basic cytocompatibility (Orive et al. 2025). The Gel facilitated improved cell adhesion and proliferation relative to the control and CIP groups, with an increase of ~120% in NIH3T3, ~115% in HaCaT, and ~120% in L929, suggesting that the hydrogel matrix provided a favorable surface for cell growth.

Adding MoO₂ to the hydrogel matrix makes the cells to proliferate slightly lower than the gel, with values of ~100% in NIH3T3, ~95% in HaCaT, and ~92% in L929, probably because the nanoparticles induced mild cellular stress; however, substantial cell coverage was observed across all the cell lines (Tian et al. 2023). On the other hand, Gel@FGF-2 exhibited the highest proliferation, with dense, confluent cell layers and strong green fluorescence in NIH3T3 (~160%), HaCaT (~155%), and L929 cells (~210%). These findings are consistent with the established pro-proliferative activity of FGF-2. The composite Gel@MoO₂ + CIP + FGF-2 under non-irradiated conditions maintained high proliferation levels of ~140% in NIH3T3, ~135% in HaCaT, and ~155% in L929, although it was slightly lower than FGF-2 alone. This indicates that FGF-2 effectively counteracts the proliferation-inhibitory effects of CIP and MoO₂. After NIR irradiation, there was a slight drop in cell density in the Gel@MoO₂ + CIP + FGF-2 group, decreasing to ~130% in NIH3T3, ~125% in HaCaT, and ~140% in L929. This is in line with photothermal-induced cellular stress (Wang et al. 2024). However, proliferation was still much higher than in the CIP-only and MoO₂-only groups. Overall, these results demonstrate that FGF-2 plays a crucial role in promoting cell proliferation across multiple cell types, whereas CIP exhibits a stronger inhibitory effect; moreover, the multifunctional hydrogel system enables balanced integration of antimicrobial activity and regenerative potential.

#### 3.4.5. Intracellular ROS scavenging and antioxidant activity evaluation

Intracellular ROS levels were evaluated using the DCFH-DA fluorescent probe; oxidative stress was induced by H₂O₂, as described in Section 2.7.4. Subsequently, all therapeutic formulations were evaluated for their antioxidant activity, as described in Section 2.8.3. In all three cell lines (NIH3T3, HaCaT, and L929), the untreated control groups exhibited negligible fluorescence, with intensities of ~0.5–1.0 a.u., indicating very low basal ROS generation (Figures SI 7A and B). When the cells were exposed to H₂O₂, a pronounced increase in green fluorescence intensity was observed, reaching ~20 a.u. in NIH3T3, ~17 a.u. in HaCaT, and ~32 a.u. in L929 cells, confirming successful intracellular ROS induction, as evidenced by the oxidation of DCFH-DA to fluorescent DCF (Liang et al. 2023). The cells treated with CIP after H₂O₂ induction showed only a marginal reduction in fluorescence, with values of ~8 a.u. (NIH3T3), ~7 a.u. (HaCaT), and ~15 a.u. (L929), which suggests that it exhibits limited antioxidant activity or ROS scavenging.

Treatment with the blank hydrogel (Gel/H₂O₂) resulted in a modest decrease in intracellular ROS, reducing fluorescence to ~6 a.u. in NIH3T3, ~5 a.u. in HaCaT, and ~12 a.u. in L929, likely due to partial physical protection and restricted ROS diffusion, although considerable oxidative stress remained (Li et al. 2023). In contrast, Gel@MoO_2_ markedly reduced DCF fluorescence across all the cell lines (~2 a.u. (NIH3T3), ~1 a.u. (HaCaT), and ~2 a.u. (L929)), indicating that they neutralized ROS, which was consistent with MoO₂'s redox activity. After oxidative stress, FGF-2 treatment also caused a clear decrease in intracellular ROS (~3–4 a.u. in NIH3T3 and HaCaT and ~7 a.u. in L929), reflecting its cytoprotective role in promoting cellular recovery under oxidative conditions. The Gel@MoO₂ + CIP + FGF-2 composite exhibited the most significant reduction in ROS levels under non-irradiated conditions, such as ~1.0 a.u. in NIH3T3, ~0.8 a.u. in HaCaT, and ~1.2 a.u. in L929, indicating that an enhanced antioxidant effect arises from the combined action of MoO₂ and FGF-2 (Zhao et al. 2022). When NIR radiation was applied, the fluorescence increased slightly relative to that of the non-irradiated composite (~1.5–2.0 a.u. across the three cell lines). This was probably due to photothermal modulation of the cellular redox balance. However, the ROS levels were still much lower than those in the H₂O₂-only groups (Austin et al. 2025). The DCFH-DA assay results showed that H₂O₂ can cause oxidative stress; nevertheless, the multifunctional hydrogel system, particularly formulations containing MoO₂ and FGF-2, can effectively lower intracellular ROS. This finding shows that the system could protect cells from oxidative damage in environments that are both regenerative and inflammatory.

#### 3.4.6. Biological activity of released FGF-2 following encapsulation and NIR irradiation

Scratch wound healing and CCK-8 assays were used to evaluate the effects of FGF-2 on fibroblast migration and proliferation, respectively, to study whether FGF-2 still retained its biological activity after release from the multifunctional hydrogel and multiple NIR irradiations (Figure SI 8). The scratch wound healing assay (Figure SI 8A and B) showed minimal wound closure in the control and Gel groups at 24 h, which indicated that there was no significant migration-promoting activity. As expected, treatment with free FGF-2 significantly increased the rate of wound closure, confirming its well-known ability to stimulate fibroblast migration. Importantly, fibroblasts treated with released FGF-2 (NIR−) and released FGF-2 (NIR+) also showed substantial wound closure, comparable to that of the free FGF-2 group under the tested conditions. The released FGF-2 groups showed a slight decrease in migration compared to free FGF-2, but the difference was minimal, indicating that the released growth factor was still largely able to retain its biological function after hydrogel encapsulation and subsequent NIR irradiation.

The CCK-8 assay further supported these findings (Figure SI 8C). The blank gel maintained cell viability similar to that of the control throughout the study period, indicating its good cytocompatibility. However, free FGF-2 significantly enhanced the proliferation of L929 fibroblasts at 24, 48, and 72 h. Cells treated with released FGF-2 (NIR-) and released FGF-2 (NIR+) showed similar proliferative responses, with only slight decreases compared to free FGF-2. The comparable responses of the released FGF-2 groups indicate that the biological activity of the released growth factor was largely maintained following the applied NIR treatment. Overall, the scratch wound healing and CCK-8 assay results indicated that FGF-2 could still retain the ability to promote fibroblast migration and proliferation after being released from the multifunctional hydrogel, even after repeated NIR irradiation. These results are complementary to the data of the release determined by ELISA and confirm that the hydrogel not only provides a sustained release of FGF-2 but also maintains its biological function, thus supporting its potential application in stimulating the regeneration of wound tissue.

### 3.5. *In vivo* biological studies

#### 3.5.1. *In vivo* wound healing studies in an infected excisional wound model

**Figure 7.**
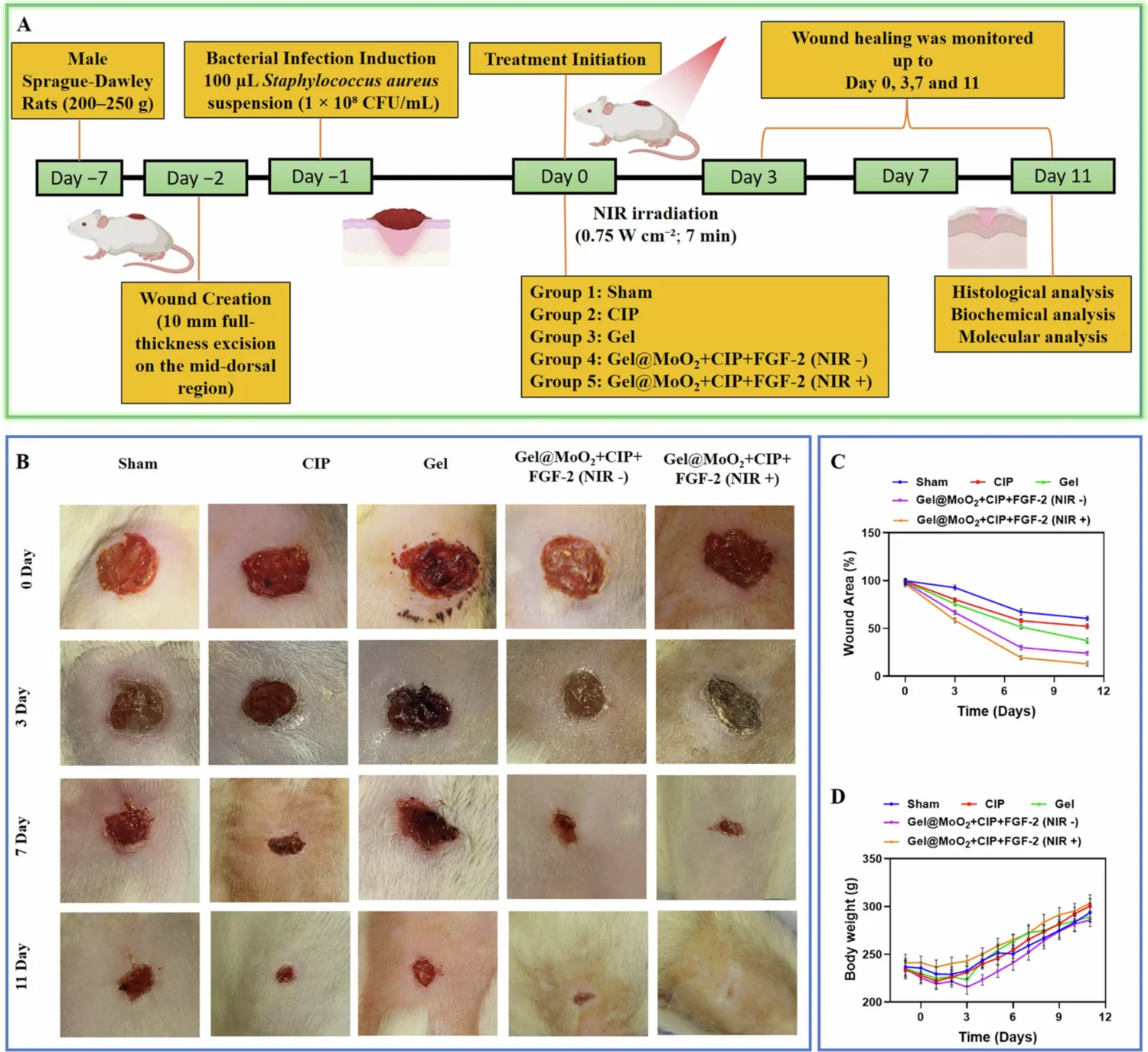
*In vivo* evaluation of antibacterial wound healing following treatment with Gel@MoO₂ + CIP + FGF-2 under NIR irradiation. (A) Schematic illustration of the infected wound model and treatment protocol. (B) Representative images of wound healing on Days 0, 3, 7, and 11. (C) Quantitative analysis of wound closure (%). (D) Body weight changes during the 11-day treatment period. For the NIR-treated group, irradiation was performed using an 808 nm laser at 0.75 W cm^−2^ for 5 min. The data are presented as mean ± SD (*n* = 5). Statistical analysis was performed using one-way ANOVA followed by Tukey's post hoc test. The exact *p*-values are presented in the figure, and statistical comparisons were performed between the indicated treatment groups, as denoted by the significance bars.

The therapeutic efficacy of the hydrogel composites was assessed using an *S. aureus*-infected full-thickness excision wound model in Sprague–Dawley rats (Figure 7A). Figure 7B shows representative images of the wounds taken on days 0, 3, 7, and 11. This figure shows that the healing process differed across the treatment groups. On day 0, all groups exhibited wounds of comparable size and morphology (~100% wound area), confirming consistent wound creation. In the sham group, wound closure was slow, with visible inflammation and incomplete epithelialization even at later time points. Quantitative analysis showed that the relative wound area remained at ~90% on day 3, ~70% on day 6, and ~60% by day 11, indicating that the body's natural healing capacity is limited during infection (Figure 7C) (Norahan et al. 2023). The CIP-treated animals showed a slight improvement compared with the sham group, with reduced exudate and gradual wound contraction to ~80% (day 3), ~55% (day 6), and ~50% (day 11). Although the bacterial burden was partially controlled, persistent scabbing and delayed closure remained evident, indicating that antibacterial treatment alone was inadequate for complete tissue regeneration (Ding et al. 2022). The blank hydrogel group showed better wound healing than the sham group, and the results were comparable to those of the CIP groups. Specifically, the wound areas decreased to ~75% on day 3, ~50% on day 6, and ~40% on day 11, accompanied by healthier granulation tissue formation. These findings suggest that the hydrogel's moist and protective microenvironment helps tissue repair. Notably, wounds treated with Gel@MoO₂ + CIP + FGF-2 under non-irradiated conditions demonstrated improved wound contraction and earlier scab resolution (~65% on day 3, ~30% on day 6, and ~25% by day 11), highlighting the improved effects of antibacterial activity, antioxidant protection, and growth factor-mediated tissue regeneration. The Gel@MoO₂ + CIP + FGF-2 (NIR+) group showed the most pronounced macroscopic wound closure among the treatment groups. By day 11, the wound had almost completely closed, with minimal scarring, and the skin had almost completely re-epithelialized. These findings indicate that NIR-triggered photothermal activation enhances treatment efficacy by clearing bacteria and altering the local wound microenvironment (Yang et al. 2025). Although the incorporation of growth factors and nanomaterials may increase the initial formulation cost, this can be mitigated through dose optimization, localized delivery, and scalable synthesis strategies. Importantly, improved healing efficiency and reduced treatment duration may lower the overall treatment cost burden.

#### 3.5.2. Quantitative wound healing and systemic safety assessment

Quantitative analysis of wound area reduction (Figure 7C) confirmed the visual observations, with the NIR-treated composite group exhibiting the fastest decrease in the relative wound area over time, followed by the non-irradiated composite, gel, CIP, and sham groups. These findings demonstrate that the multifunctional hydrogel system enhanced wound closure under the tested infection-wound conditions (Chen et al. 2024). Throughout the treatment period, body weight was monitored (Figure 7D) to assess systemic biocompatibility. All groups showed a slight initial decrease in body weight during the early inflammatory phase (~220–235 g around day 3), followed by steady recovery and weight gain. By day 12, body weights reached ~285 g (sham), ~290 g (CIP), ~295 g (Gel), ~300 g (Gel@MoO₂ + CIP + FGF-2 (NIR−)), and ~305 g (Gel@MoO₂ + CIP + FGF-2 (NIR+)), with no significant differences observed among the groups. This finding indicates favorable systemic tolerance and the absence of treatment-related toxicity.

#### 3.5.3. *In vivo* antibacterial efficacy evaluation

**Figure 8.**
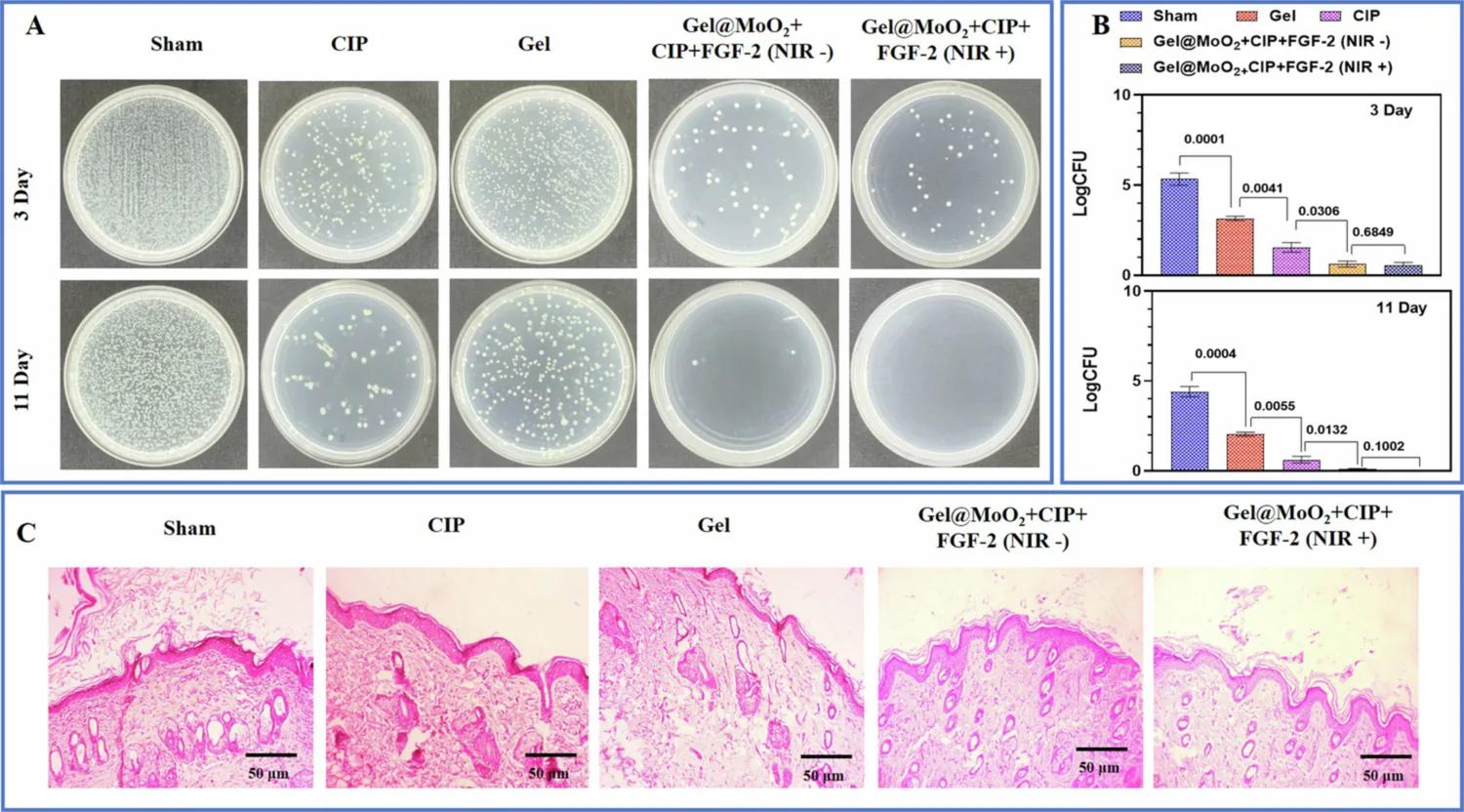
*In vivo* antibacterial efficacy and histological evaluation of infected wounds following treatment with Gel@MoO₂ + CIP + FGF-2. (A) Representative images of S. aureus colonies recovered from wound tissues on Days 3 and 11 following treatment with Sham, CIP, Gel, Gel@MoO₂ + CIP + FGF-2 (NIR−), and Gel@MoO₂ + CIP + FGF-2 (NIR+). For the NIR-treated group, irradiation was performed using an 808 nm laser at 0.75 W cm^−2^ for 5 min. (B) Quantitative analysis of the bacterial burden expressed as log₁₀ CFU on Days 3 and 11. The data are presented as mean ± SD (*n* = 5). Statistical analysis was performed using one-way ANOVA followed by Tukey's post hoc test. Exact *p*-values are presented in the figure, and statistical comparisons were performed between the indicated treatment groups, as denoted by the significance bars. (C) Representative hematoxylin and eosin (H&E)-stained histological images of wound tissues collected on Day 11, showing epidermal regeneration and dermal remodeling following the different treatments.

*In vivo* antibacterial efficacy was further assessed by enumerating CFU from wound swabs collected on days 3 and 11 (Figure 8A and B). On day 3, the sham group exhibited high bacterial loads (5.33 ± 0.40 log CFU), while CIP and hydrogel treatments resulted in partial reductions of 1.55 ± 0.30 log CFU and 3.21 ± 0.20 log CFU, respectively. In contrast, wounds treated with Gel@MoO₂ + CIP + FGF-2 showed substantially lower CFU counts (0.62 ± 0.20 log CFU), with the NIR-irradiated group displaying the lowest bacterial burden among the composite-treated groups (0.56 ± 0.10 log CFU), which is consistent with reports demonstrating enhanced bacterial clearance through photothermal or nanozyme-assisted hydrogel systems (Pahlevani et al. 2025; Chegini et al. 2025). By day 11, residual bacterial colonies were still detectable in the sham group (4.40 ± 0.40 log CFU), but the bare Gel groups exhibited colonies of 2.03 ± 0.30 log CFU. While CIP-treated wounds showed further reduction to 0.62 ± 0.20 log CFU. Notably, the Gel@MoO₂ + CIP + FGF-2 group exhibited nearly complete bacterial clearance, with counts decreasing to 0.11 ± 0.05 log CFU without irradiation and below the detection limit (<0.3 log CFU) under NIR irradiation. These results closely correlate with the observed wound healing outcomes, highlighting the importance of effective bacterial clearance for tissue regeneration (Wei et al. 2023; Li et al. 2024). Although the current investigation utilized an infection model using *S. aureus* that is commonly used and clinically relevant to test wound infection treatments, is a relatively simple system and does not entirely reflect the complexity seen in chronic wounds, particularly in relation to polymicrobial infection, diabetes mellitus, or poor healing environments. Further research efforts will be directed towards developing a polymicrobial or diabetic wound infection model to better mimic a preclinical setting.

#### 3.5.4. Histopathological evaluation of tissue regeneration and wound remodeling

Histopathological evaluation of wound tissues harvested on day 11 (Figure 8C) further corroborated the macroscopic and quantitative healing outcomes (Liu et al. 2024). In the sham group, the epidermis remained discontinuous and irregular, accompanied by loosely organized dermal collagen, persistent inflammatory cell infiltration, and incomplete tissue remodeling, indicating delayed healing under infection conditions (Pranantyo et al. 2024). The CIP-treated group showed partial re-epithelialization with a moderate reduction in inflammation; however, the epidermal layer was thin and uneven, and the collagen fibers remained poorly aligned, suggesting that antibacterial treatment alone was insufficient for full skin regeneration. In the Gel group, improved epithelial continuity and enhanced granulation tissue formation were observed, along with moderate collagen deposition, reflecting the beneficial role of a moist and protective wound microenvironment. Notably, wounds treated with Gel@MoO₂ + CIP + FGF-2 without NIR irradiation exhibited well-formed stratified epidermis, reduced inflammatory infiltrates, and denser, more organized collagen fibres, indicating accelerated tissue repair driven by combined antibacterial, antioxidant, and growth factor effects. Notably, the Gel@MoO₂ + CIP + FGF-2 (NIR+) group exhibited a near-normal skin architecture characterized by complete re-epithelialization, thick, uniform epidermal layers, well-aligned collagen bundles, and partial restoration of skin appendages, with minimal residual inflammation. These histological features closely resemble healthy skin morphology and confirm that NIR-triggered photothermal activation markedly enhances bacterial clearance, tissue remodeling, and regenerative processes, thereby producing superior wound healing outcomes (Wang et al. 2022; Sparks et al. 2022). The improved histological outcomes accord with the reduced inflammatory burden and the enhanced regenerative microenvironment provided by the hydrogel system.

#### 3.5.5. *In vivo* antioxidant activity evaluation

To further elucidate the therapeutic mechanism, oxidative stress markers in wound tissues were analyzed by measuring malondialdehyde (MDA) levels and the activities of the antioxidant enzymes superoxide dismutase (SOD) and catalase (CAT) (Figure SI 9A). The sham group exhibited the highest MDA levels (~8.5–9.0 nmol/mg protein), indicating severe lipid peroxidation and oxidative damage induced by persistent infection. CIP treatment moderately reduced MDA level to ~6.5–7.0 nmol/mg protein, which is consistent with partial bacterial clearance but limited regulation of oxidative stress. The plain Gel group showed a further reduction in MDA content (~5.0–5.5 nmol/mg protein), which was likely attributable to physical protection of the wound surface and attenuation of inflammatory stimuli. In contrast, wounds treated with Gel@MoO₂ + CIP + FGF-2 demonstrated a pronounced decrease in MDA levels, reaching ~3.0–3.5 nmol/mg protein under non-irradiated conditions. The NIR-irradiated composite group exhibited the lowest MDA values (~2.0–2.5 nmol/mg protein), reflecting effective suppression of oxidative damage. This marked reduction can be attributed to the redox-active MoO₂ nanozyme combined with enhanced infection control under photothermal activation.

Consistent with these findings, SOD activity was lowest in the sham group (~25–30 U/mg protein) and increased progressively in the CIP (~40–45 U/mg protein) and Gel (~50–55 U/mg protein) groups. SOD activity was significantly higher in the Gel@MoO₂ + CIP + FGF-2-treated wounds than in the corresponding treatment groups, as indicated in Figure SI 9A, reaching ~75–80 U/mg protein in the non-irradiated group and ~90–95 U/mg protein following NIR irradiation. A similar trend was observed for CAT activity, which increased from ~18–20 U/mg protein in the sham group to ~24–26 U/mg protein with CIP and ~33–36 U/mg protein in the hydrogel group. The highest CAT activities were recorded in the composite-treated wounds, reaching ~55–58 U/mg protein without irradiation and ~65–70 U/mg protein under NIR irradiation. The progressive elevation of SOD and CAT activities, together with the substantial reduction in MDA levels, indicates the restoration of endogenous antioxidant defenses and efficient detoxification of reactive oxygen species. Collectively, these biochemical results demonstrate that the multifunctional Gel@MoO₂ + CIP + FGF-2 hydrogel effectively alleviated oxidative stress in infected wounds, thereby creating a favorable microenvironment for tissue repair, particularly when combined with NIR-triggered photothermal activation.

#### 3.5.6. Modulation of the Nrf2/Keap1 antioxidant signaling pathway

To further investigate the molecular basis for the antioxidant activity observed in the hydrogel-treated wounds, the expression levels of Nrf2, Keap1, and HO-1 were evaluated via qRT‒PCR (Figure SI 9B). Hydrogel-treated wounds showed increased Nrf2 and HO-1 expression and reduced Keap1 expression relative to the corresponding comparison groups, as shown in Figure SI 9B. The Gel@MoO₂ + CIP + FGF-2 (NIR−) group showed higher Nrf2 and HO-1 expression than the Gel and CIP groups, while the NIR+ group exhibited the highest Nrf2 and HO-1 expression and the lowest Keap1 expression. The Nrf2/Keap1 pathway is one of the key regulators of the cellular antioxidant response. Under oxidative stress conditions, Nrf2 dissociates from Keap1 and translocates to the nucleus, where it induces the expression of antioxidant and cytoprotective genes such as HO-1. The upregulation of Nrf2 and HO-1 expression and downregulation of Keap1 observed in the present study indicate the activation of endogenous antioxidant defense mechanisms after hydrogel treatment. All the results obtained are consistent with the observations in terms of high activities of SOD and CAT, low content of MDA, and decreased accumulation of ROS based on intracellular ROS assays. The increased delivery of treatment and modification of the microenvironment within the wounds in the NIR-activated group led to easier management of the oxidative stress levels, leading to a higher level of activation recorded. In general, the reduction in oxidative stress by the hydrogels is partly due to the activation of Nrf2/Keap1/HO-1 signaling pathways.

#### 3.5.7. *In vivo* gene expression analysis related to inflammation and tissue regeneration

To clarify the molecular basis of the improved wound healing observed *in vivo*, the expression of inflammation, angiogenesis, and matrix-remodeling-related genes was analyzed via RT-qPCR (Figure SI 10). The sham group exhibited the highest expression of pro-inflammatory cytokines, with IL-6 and TNF-α levels normalized to ~1.0-fold, indicating sustained inflammatory signaling in untreated infected wounds. CIP treatment moderately reduced IL-6 and TNF-α expression to ~0.8–0.85-fold, while further suppression was observed in the plain Gel group (~0.65–0.75-fold). In contrast, wounds treated with Gel@MoO₂ + CIP + FGF-2 showed a pronounced downregulation of inflammatory markers, with IL-6 and TNF-α decreasing to ~0.40–0.45-fold under non-irradiated conditions and reaching the lowest levels (~0.25–0.30-fold) following NIR irradiation. This marked reduction in inflammatory gene expression is consistent with effective bacterial clearance and attenuation of oxidative stress.

Similarly, genes associated with angiogenesis and tissue repair were progressively upregulated with advanced treatments. FGF-2 expression increased from ~1.0-fold (sham) to ~1.3-fold (CIP) and ~1.6-fold (Gel), while higher expression levels were observed in the composite-treated groups, with the NIR+ group showing the highest values. A similar trend was observed for VEGF, which increased from ~1.1-fold in the sham group to ~1.4-fold (CIP), ~1.9-fold (hydrogel), ~2.8–3.0-fold (NIR−), and ~3.5–3.7-fold (NIR+), indicating pro-angiogenic activity in the composite-treated wounds.

Changes in MMP-9 and Col-1 expression further reflected matrix remodeling. The MMP-9 levels were highest in the sham group (~1.0-fold) and decreased gradually with treatment, reaching ~0.5-fold in the non-irradiated composite group and ~0.3-fold under NIR irradiation, suggesting controlled extracellular matrix degradation. In contrast, Col-1 expression increased from ~1.0-fold (sham) to ~1.3-fold (CIP) and ~1.8-fold (hydrogel), with a marked upregulation in the composite groups (~3.0-fold for NIR− and ~3.6–3.8-fold for NIR+), indicating enhanced collagen deposition and matrix maturation. Overall, the RT-qPCR results demonstrated that Gel@MoO₂ + CIP + FGF-2 treatment effectively suppressed inflammatory signaling while providing a favorable environment for angiogenesis, extracellular matrix stabilization, and collagen synthesis. NIR irradiation further amplifies these effects, providing a molecular explanation for the superior wound closure, reduced oxidative stress, and advanced tissue remodeling observed *in vivo*.

Despite the promising therapeutic effects demonstrated by this study, some limitations should be acknowledged. However, despite the increased expression of VEGF and FGF-2, which suggested the establishment of a pro-angiogenic microenvironment, a direct evaluation of neovascularization using vascular markers such as CD31 was not performed. The anti-inflammatory, antioxidant, and regenerative effects observed were confirmed by qRT-PCR, ELISA, ROS analysis, SOD, CAT, and MDA measurements, migration assays, and histological analysis. Furthermore, the activation of the Nrf2/Keap1/HO-1 pathway offered further mechanistic rationale for the antioxidant activity of the hydrogel system. However, pathway-specific studies of greater scope, such as Western blotting, immunohistochemistry, and inhibitor-based studies, would provide deeper mechanistic insight.

Thermal safety was assessed under selected NIR irradiation conditions (0.75 W cm^−2^, 5 min), and no detectable tissue damage was observed; however, the present study did not systematically evaluate the effects of repeated NIR treatment on surrounding tissues over an extended period. Although the hydrogel demonstrated reproducible photothermal responses during repeated NIR on–off cycles and the biological activity of released FGF-2 was maintained after irradiation, repeated clinical treatment may involve cumulative thermal exposure that could vary with tissue thickness, perfusion, wound condition, and irradiation frequency. Therefore, future studies should optimize the frequency and duration of NIR treatment and evaluate the cumulative thermal effects and local tissue safety during repeated irradiation.

The long-term safety and fate of MoO₂ nanoparticles also remain to be established. Although the present study demonstrated favorable short-term cytocompatibility and wound tolerance, it did not determine the tissue distribution, persistence, clearance, or potential accumulation of MoO₂ following local administration. In addition, the hydrogel degradation assessment was based on overall mass loss and did not chemically identify or quantify the degradation products generated during degradation or determine the fate of the MoO₂ component within the degrading matrix. Future studies should therefore characterize the degradation products and evaluate their local and systemic safety, together with the retention and clearance of MoO₂ nanoparticles, using appropriate long-term histopathological and analytical assessments.

From a translational perspective, the multifunctional nature of the formulation also presents several practical considerations, including reproducible incorporation of MoO₂, CIP, and FGF-2, preservation of FGF-2 biological activity, controlled release of the therapeutic components, sterilization, storage stability, batch-to-batch consistency, and scalable manufacturing. These parameters, together with pharmacokinetic and pharmacodynamic profiles, need to be established under clinically relevant conditions before advancement toward clinical application.

Moreover, the therapeutic efficacy of the developed hydrogel was evaluated using an acute MRSA-infected excisional wound model, which, although widely accepted for the preclinical evaluation of antibacterial wound dressings, does not completely mimic the complicated pathological features of chronic wounds, including sustained inflammation, inhibited angiogenesis, and delayed tissue regeneration. Further studies in clinically relevant chronic wound models, especially in diabetic infected wound models, are warranted to further validate its long-term therapeutic efficacy and clinical translational potential. The relatively small sample size (*n* = 5 per group) might also constrain the generalisability of the findings. In addition, although no significant weight loss, mortality, or abnormal behavioral changes were observed, detailed systemic biosafety assessments, including organ histopathology, hematological analysis, serum biochemistry, and long-term toxicity evaluations, were not performed. Thus, the present work should be considered a proof-of-concept study, and comprehensive long-term safety, pharmacokinetic, degradation, manufacturing, and regulatory evaluations in larger and clinically relevant models are needed before clinical translation.

## 4. Conclusion

This study presents a sprayable, stimuli-responsive NSC/PF127 hydrogel integrating MoO₂ nanoparticles, CIP, and FGF-2 for the treatment of chronic infected wounds. The hydrogel undergoes rapid thermally induced gelation and enables pH-responsive drug release together with NIR-triggered photothermal activation. The multifunctional system exhibits effective antibacterial and antibiofilm activity, reduces oxidative stress, and maintains good cytocompatibility while supporting cell migration and proliferation. In an MRSA-infected full-thickness wound model, the composite hydrogel significantly accelerated wound closure, reduced the bacterial burden, restored the antioxidant balance, and promoted pro-angiogenesis and collagen remodeling, with NIR irradiation further enhancing therapeutic efficacy. Gene expression analyses confirmed the coordinated suppression of inflammatory mediators and the upregulation of angiogenic and extracellular matrix-related genes. Collectively, these results demonstrate that integrating photothermal nanozymes, antibiotic delivery, and growth factor signaling within a single hydrogel platform offers an effective strategy for promoting regeneration in infected wounds and supports further development of multifunctional nanobiomaterials for wound-healing applications. Overall, the developed multifunctional sprayable hydrogel represents a promising therapeutic platform for infected wound healing and provides a strong foundation for future investigations in clinically relevant chronic wound models.

## Supplementary Material

Author_Checklist_Arrive_guidelines.pdf

## Data Availability

The data will be provided upon request. No data have been omitted or retained from inclusion in this published article.
